# Implementation of the visual aesthetic quality of slope forest autumn color change into the configuration of tree species

**DOI:** 10.1038/s41598-021-04317-1

**Published:** 2022-01-20

**Authors:** Yanxia Mu, Wenyue Lin, Xiuli Diao, Zhe Zhang, Jin Wang, Zijing Lu, Wencheng Guo, Yu Wang, Chunxiang Hu, Changyou Zhao

**Affiliations:** 1 Southwest Research Center for Landscape Architecture Engineering, College of Landscape Architecture and Horticulture, Southwest Forestry University, Kunming, 650224 China; 2 Yunnan Province Management and Protection Agency of Jiaozi Mountain National Nature Reserve, Kunming, 651515 China

**Keywords:** Environmental social sciences, Psychology and behaviour, Sustainability

## Abstract

Urban expansion leads to changes in the visual aesthetic quality and ecological degradation of the surrounding slope forest landscapes. Color is a crucial visual element to examine when viewing this large-scale slope forest landscape from a long distance. This is particularly true for the autumn color of slope forest, which is very attractive to the public. An exploration of the relationship between the change in color of a natural slope forest and its visual aesthetic quality enables the implementation of the configuration of superior aesthetic tree species. Therefore, it can provide aesthetic rules and a reference to configure local tree species to support their visual aesthetic quality, ecological sustainability and native biodiversity restoration in a local urban slope forest. However, such research is critically lacking. This study investigated the visual aesthetic quality of the color dynamics of a natural slope forest in Jiaozi Mountain, China in the autumn. We analyzed both the composition of tree species and the changes in color for each species of tree in nine forest sites that exhibited superior visual aesthetic quality. The results showed that the forests with superior visual aesthetic quality were more green, red, and yellow, had moderately higher saturation and value, more obvious color contrast, and diverse colors with primary and secondary contrast. Diverse and balanced color patches or a dominant color patch contrasted by many small patches with interspersed color components also highlighted the superior visual aesthetic quality of slope forest features. Different combinations of color features can result in high visual aesthetic quality. The 84 tree species in the superior visual aesthetic quality forests primarily displayed 10 types of color changes that varied as green, yellow, blue, red, withered yellow, withered red and gray.

## Introduction

As urban populations and areas increase, slope forests within or around cities become increasingly important for the well-being of residents and urban sustainability^1–3^. They have become a focus of global attention as a nature-based solution^4–7^. However, these slope forests in cities have been disturbed with urban expansion^8–10^. In particular, the mountainous areas of China comprise more than two-thirds of the area of land in this country^11^. The rapid expansion and construction of many mountainous cities in China have caused damage to the slope forests inside and around them, resulting in a decrease in landscape quality^12,13^, ecological stability and biodiversity^14,15^. Thus, China’s national strategy is to aggressively work toward improving the quality of urban slope forest landscape, scientifically select tree species, and adjust the pattern of distribution of tree species, so that residents can see the beautiful slope forest landscape from the city^16^. Moreover, it emphasizes their biodiversity and ecological sustainability^10,16^. Therefore, it is highly important and urgent to study ways to improve the quality of landscape and promote the ecological benefits of urban slope forests through the selection and configuration of tree species^17,18^.

A large-scale urban slope forest landscape may be primarily appreciated from a relatively long distance, since it may be difficult for residents to walk into it and experience the comprehensive visual, auditory, taste and touch. Therefore, improving its visual aesthetic quality is the key to improving landscape quality^19–22^. Visual aesthetic quality refers to the perceptual evaluation of landscape elements and features, such as color, movement, and pattern, by a human observer^23,24^. However, when a slope forest landscape is viewed from a relatively long distance, observers may not be able to fully see the details in forest, such as the shape of individual trees, but instead will see the overall image. Cui et al.^25^ observed that when people watch the landscape, color perception comprises 80% in the first 20 s, while the perception of other elements, such as shape and texture, comprises 20%. After 2 min, color is 60%, and the others are 40%. After 5 min, the ratio is 50/50 and will continue in that manner. Hence, color is a crucial visual landscape element and intensely stimulates the visual perception and substantially affects the visual aesthetic quality of a slope forest landscape. It can be a crucial factor that enhances the visual aesthetic quality of a slope forest^20,22,26–28^.

In fact, the color of the slope forest strongly appeals to the public, particularly in the autumn^29–31^. In parts of the northeastern United States and southeastern Canada, tourism to view fall leaves is estimated to comprise $400 million^32^. In Japan, many forest tourist attractions will hold autumn leaf coloring festivals to attract tourists. The opening days are determined based on the duration of autumn leaves to maximize tourism revenue^33^. In China, slope forest attractions engage a large number of tourists during the colorful autumn period, driving the forest tourism industry^19,31,34,35^. Therefore, the exploration and optimization of the changes in autumn color of slope forests is an important step toward improving the visual aesthetic quality of slope forests^19,31^.

In previous research on plant color, photos of flowers, fruit, and leaves were collected throughout the year to study the seasonal changes in color and determine those time points when the plant color is perceived to be the most beautiful^36–39^. However, in the field of slope forest color research, few studies have been formulated an effective research method that dynamically evaluates the progression of color changes during a certain season or even throughout the year.

Existing studies that examine the visual aesthetic quality of slope forest colors have been examined from three different aspects. The first involves monitoring by humans. This type of research entails selected human indicators of visual perception, such as eye-tracking visual indicators (fixation frequency, average fixation time, and saccade frequency)^27,40,41^. In addition, other physiological indicators, such as brain waves, skin conductivity, blood pressure, respiratory rate and electroencephalograms (EEGs), were used to evaluate the impact of different forest colors on human subjects^37,42,43^. Psychological associations and symbols^44^ and other psychological indicators from various psychological scales have also been examined^45,46^. The second aspect relates to the subjective aesthetic awareness of the public. This is discerned by evaluating the perceived beauty of forest colors in different seasons. By analyzing the characteristics of color types, and color patches among other aspects, researchers have summarized the principles of configuration of forest color^20,34,47,48^. The third aspect relates to the adjustment of forest color based on the evaluation of the color of slope forest to improve the visual aesthetic quality^19,49^. While there have been many studies^20,27,34,37,42,44,45,47,48^ from the first and second aspects, the third aspect has not been studied in-depth, and in particular, research on how to create ideal color combinations is lacking.

Kunstler et al.^50–52^ examined the structural patterns of tree communities from an ecological perspective, placing their emphasis on the competition and harmony of interspecies relationships. Additionally, silviculture management technology has been used to maintain complex tree stand structures^53^. Other studies^54,55^ have examined the patterns of configuration of tree species using the theory of color harmony. While Zhang et al.^19^ and Wang et al.^49^ focused on aesthetic analyses of slope forest colors, attaching importance to analytical methods and processes, they only proposed macro color configuration principles and suggested the selection of a few tree species. To our knowledge, no studies have translated the results of a visual evaluation of the aesthetics of forest color into the selection and configuration of specific tree species.

In addition, the sustainability of the forest ecosystem and biodiversity must also be considered when selecting and configuring tree species to improve the visual aesthetic quality of urban slope forest^10,16^. However, the number of existing urban green tree species in China is still small^56^ and includes many exotic ornamental species^10,16^. These species may be beautiful but are not necessarily suitable for large-scale planting in urban slope forests because they may not be adapted to local ecologically climatic conditions or require costly maintenance and management^57,58^. Numerous studies^59–61^ have demonstrated advantages in ecological sustainability and landscape aesthetics of the use of native species, which have the potential to be planted on a large-scale in urban slope forests. However, the remnant slope forest compositions of many cities are primarily artificial pure forest^62,63^, and the species of native trees are considered too limited to fulfil long-term and sustainable forest ecosystem and biodiversity^64–66^. Most of the research that led to that conclusion has proposed few solutions^64,67^. To effectively influence healthy slope forest management, an assessment of the ornamental characteristics of local native tree species and configuration in natural slope forests can provide information that supports the restoration and development of slope forest biodiversity in urban areas using native trees^17,18,68,69^. However, such studies are rare.

The issues described above lead to the following questions that merit further study:

1. How does the natural slope forest color and corresponding visual aesthetic quality change during the autumn foliage period?
2. What is the relationship between the color of the natural slope forest and the corresponding visual aesthetic quality?
3. What are the color combination characteristics of superior visual aesthetic quality in natural slope forest landscapes?
4. What are the compositions of tree species and the changes in color in each species in the natural slope forest sites that exhibited superior visual aesthetic quality?

This study attempts to tackle these issues. In addition, we attempt to formulate strategies to select and configure specific tree species to optimize slope forest colors based on the results. Our goal is intended to provide information of the visual aesthetic quality of in variation of autumn color in natural slope forests and explore the configurations of native tree species with superior changes in color. This can support the regulation and improvement of slope forest management for optimal aesthetic appeal, ecosystem sustainability and native biodiversity restoration in urban regions.

The hypotheses of this study are as follows:

1. The color of natural slope forests and the corresponding visual aesthetic quality change synchronously during the period of change of autumn color.
2. The different color characteristics of natural slope forests lead to varying visual aesthetic quality.
3. The natural slope forest landscapes that have superior visual aesthetic quality have different color combination characteristics.
4. There are differences and similarities in the composition of tree species with superior visual aesthetic quality and color changes of each species in the natural slope forests.

## Materials and methods

### Study area

**Figure 1:**
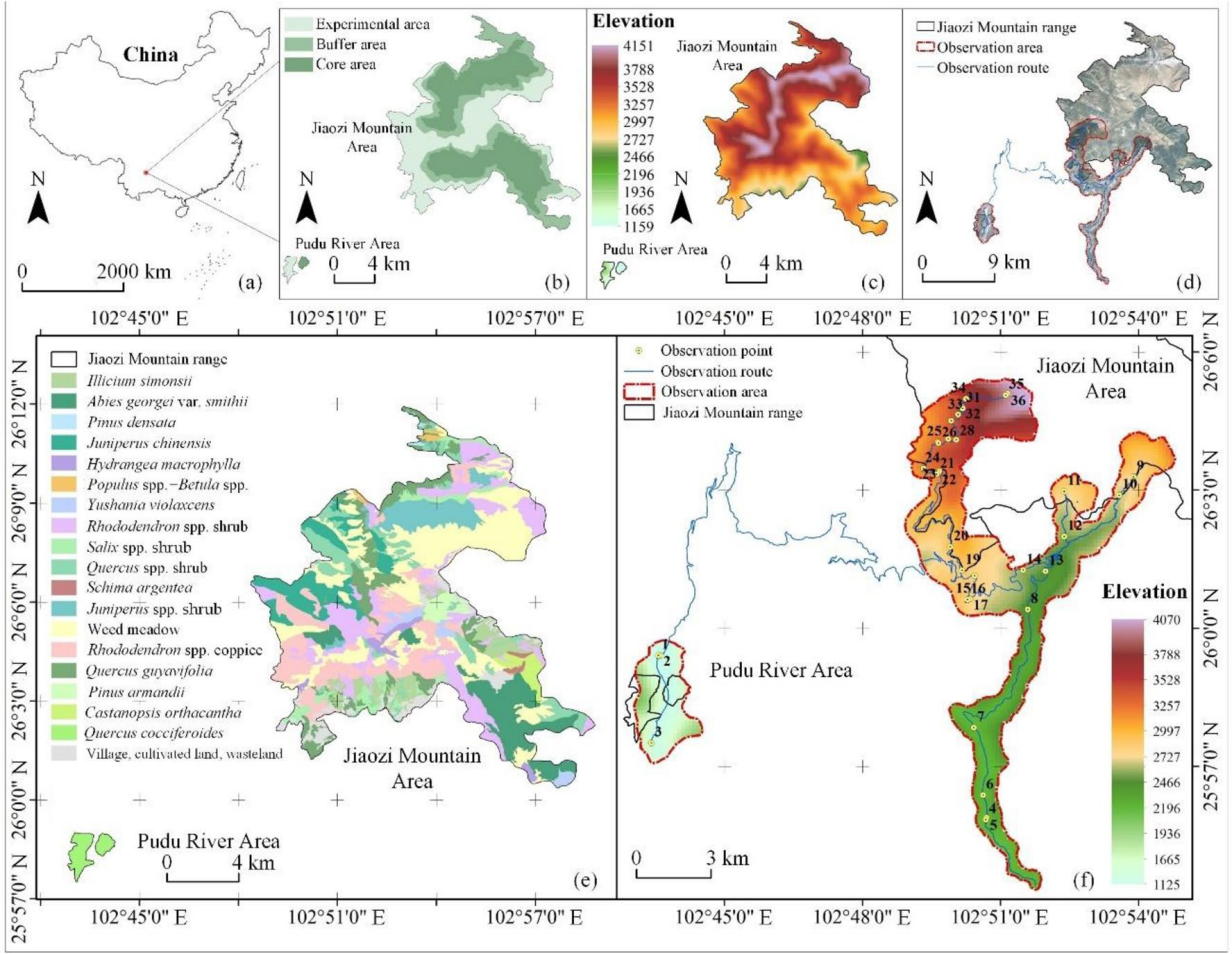
(**a**) The location in China of the study site, Jiaozi Mountain National Nature Reserve. (**b**) The protection classification within the Reserve. (**c**) Elevation distribution of Jiaozi Mountain. (**d**) Vegetation type distribution on Jiaozi Mountain. (**e**) Main vegetation types within the Reserve. (**f**) The study area boundaries and observation route.

Jiaozi Mountain National Natural Reserve is located in central Yunnan Province, China (102° 48′ 21″–102° 58′ 43″ E, 26° 00′ 25″–26° 11′ 53″ N). This area is regarded as one of the world’s centers of biodiversity^70^. It has a total area of 16,456 hectares and consists of two parts: the Jiaozi Mountain area and the Pudu River area (Fig. 1a,b). The surrounding area has mountains and canyons with elevations that range from approximately 1100 to 4300 m. Therefore, the terrain undulates dramatically (Fig. 1c), and the climate (Table S1) and soil (Table S2) have significantly vertically differentiated. Those various environmental conditions have created thriving and diverse natural types of forest vegetation on Jiaozi Mountain (Fig. 1e, Table S3)^70^. Among them are species of native subalpine forest deciduous trees with leaves that turn colors in the autumn, such as poplar, maple and birch, and evergreen tree species, such as fir, oak and cypress. Together, they change colors abundantly in the autumn. Therefore, we selected this region for our research. Because the Reserve is composed of experimental and entrance-restricted core and buffer areas (Fig. 1b), our study was primarily contained within the experimental areas of the eastern part of the mountain, as well as the Pudu River area (Fig. 1d). Those are also the main scenic areas in the reserve, and they attract many tourists who enjoy natural slope forest landscapes.

### Selection and measurement of observation points

**Figure 2:**
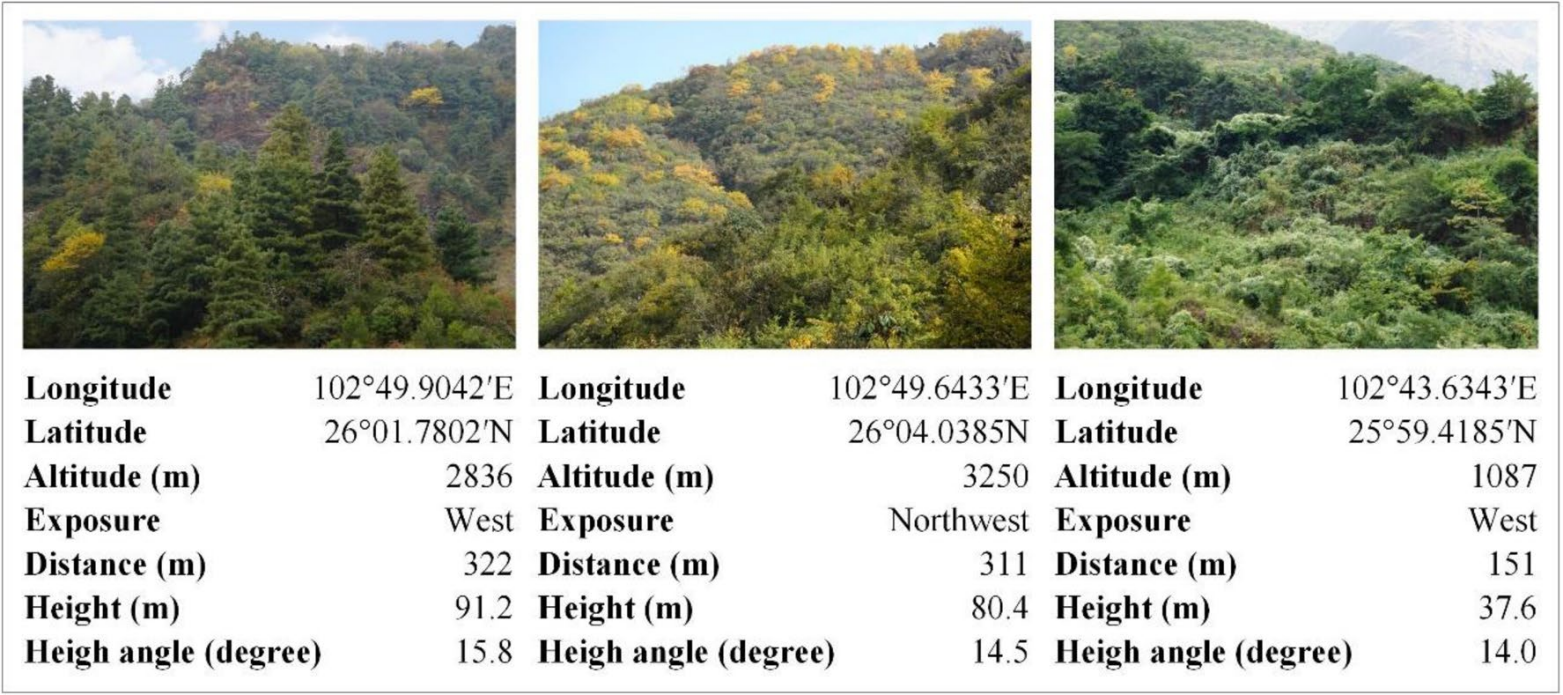
Representative photos of target forests taken at the observation points. Distance and height are between an observation point and its targeted forest area. The height angle is the angle between the horizontal plane and the line of direction from an observation point to the center of the target slope forest.

We selected observation points along the observation route (Fig. 1f) based on the diversity of the forest landscapes: (1) Since the vegetation on Jiaozi Mountain differed vertically, observation points at altitudes of 1000–1999, 2000–2999, and 3000–3999 m were selected for the survey, so that the diversity of vertical vegetation was well-represented. (2) In addition to accounting for vertical diversity, we also considered the distribution of horizontal vegetation by following an observation route that included as many representative plant communities as possible. We also considered the following requirements for photography: (1) The observation points must have a good field of view conditions to ensure the photographs of the overall forest that did not include non-forest elements^71^. (2) In general, the distance between an observation point and the target slope forest was not too far or too close. If the distance was too close (i.e., less than 100 m), it is difficult to completely include the overall shape of mountain slope, color changes of the slope forests and the combination of relationships between patches of different tree species. If the distance was too far (i.e., farther than 600 m), it was difficult to observe the types of trees and shrubs and other forest elements, and the visibility of observation and photo clarity were unreliable^72^. Finally, we found that 200–400 m was an ideal observation distance based on these parameters. Therefore, we tried to choose the correct range of this distance for shooting. To stay safe, we sometimes had to abandon observation points that had become unsafe. During the study, we measured the latitude, longitude, altitude, slope aspect, distance, height, and observation height angle from the observation point to that point’s target forest area (as shown in Fig. 2). Ultimately, we selected 36 observation points (Fig. 1f).

We also identified the composition of tree species for each target slope forest area using a combination of methods. (1) On-site identification. We identified plant types as closely as possible by utilizing telescope magnification. In addition, we identified the type of the same plant based on its coloration at different stages of discoloration. We also compared distant plants with nearby plants under similar morphological characteristics and habitat conditions to infer the types of distant plants. (2) Identification from photos. We took detailed photographs of the plants in the target slope forest area, including photos of the plant organs. We consulted relevant experts and study data from the Jiaozi Mountain Vegetation community to identify these plants. (3) Identification based on plant specimens. We collected the stems, leaves, flowers and fruits of some plants and identified these plant types by consulting relevant experts and referring to records of the flora.

### Photographing forest color variations

Owing to the distinct 3-dimensional climate characteristics of Jiaozi Mountain, phenological color change periods differed between observation points. Thus, we need to adequately track the color change process over time to ensure a relatively consistent record of forest color change based on annual climate changes. We used the records of 5 years prior to 2019 from the Jiaozi Mountain meteorological observatory to determine the best autumn observation period^34^. We found that the 2019 autumn temperatures mirrored the climate trend of the past 5 years. Thus, the autumn forest color variations in 2019 should match those of the previous 5 years. Using those observations, we determined that mid-to-late August to the end of November in 2019 would be an appropriate observation period for our study. To track and photograph autumn discoloration, that observation period was divided into five discoloration periods: The pre-discoloration period (DV1, mid- to late August to late September), the early to middle period (DV2, early to mid-October), the middle period (DV3, late October), the late period (DV4, early to mid-November), and the end period (DV5, late November to the end of November).

We standardized our photography as follows^73^. (1) We chose sunny days with high visibility. (2) The preferred photographic time was between 3 h after sunrise and 3 h before sunset^74,75^. (3) We avoided direct sunlight. (4) We looked up and straight ahead to see the overall forest. (5) One photographer took all the photos using the same a6000 camera (Sony Corp., Tokyo, Japan) with the same settings and always repeating the same position, direction, angle, distance, and shooting mode in a horizontal format.

We also screened the photo as follow: One photo from each targeted forest area for each discoloration period was chosen^76^. During the experiment, some targeted forests in some photographic records were missing because of lasting heavy fog or snow cover. However, by the end of the study, we had obtained 120 photos that effectively reflected the changes in slope forest color.

### Evaluating the visual aesthetic quality of slope forest color

The first was the selection of evaluators. Previous social surveys in China have shown that when the sample size > 400, confidence values > 95%^19,77^*.* Therefore, for reliable evaluation, the effective collected sample size of this study was 468 evaluators. Several studies have shown that people of all ages, genders, educational backgrounds, occupations, incomes, regional origins, and professional backgrounds have universally consistent aesthetic opinions of natural scenery^78–80^. Therefore, we recruited individuals from the general public as subjects to evaluate the photos for forest color. Their genders, ages, educational and professional backgrounds, and regional origins are listed in Table S4.

We used scenic beauty estimation (SBE), an efficient and objective method that is widely used to evaluate the aesthetic quality of forest landscapes^24,31,34,81–84^, to conduct the visual evaluation of the aesthetic quality of slope forest color. Prior to evaluation, we eliminated any possible influence of sky backgrounds by replacing them in the photos of the same target forest areas with the same blue sky and white clouds. The SBE questionnaire contained the color photos of each target forest area. To evaluate the photos, we created a 7-point scale from − 3 to 3, with − 3 representing the lowest visual aesthetic quality, and 3 representing the highest visual aesthetic quality. To eliminate the primacy effect, the evaluators were first shown a preview of five demos that were similar to the test photos, and then they devised their own criteria to assign a 7-point scale score. Subsequently, each evaluator saw all 120 test photos with each photo shown only once for 6 s. Each evaluator was assigned randomly ordered photos^34,85^, thus, avoiding a reduction in the sensitivity of evaluation owing to a continuous succession of photos of the same forest.

Since each SBE value was affected by the individual aesthetic standard differences of the evaluators, direct calculation could bias the results. To eliminate that bias, we standardized the data^82^ as follows:

1 $$SBEsvi=\left(\overline{Zi}-\overline{Zi}\right)\times 100=0,$$

2 $$SBEsva=\left(\overline{Za}-\overline{Zi}\right)\times 100,$$

3 $$\overline{Z}=\frac{{\sum }_{k=1}^{n-1}\left[NOR\left(cp\right)\right]k}{k},$$

where *SBEsv* is the SBE standardized value of a certain photo; *i* is a randomly selected comparison photo; *a* is other photos; *cp* is the cumulative probability of a certain photo’s score grade, and $\overline{Z}$ is the arithmetic average of *Z*, which is the one-sided quantile value of the normal distribution of *cp*. Thus, when *cp* = 1, *cp* = 1 − 1/(2* N*) was used to calculate *Z*, and when *cp* = 0, *cp* = 1/(2* N*) was used (*N* = 468). Here, *n* is the score grade from 1 to 7 (corresponding to − 3 to 3), and *k* is the cumulative count.

After the SBE values were standardized, we analyzed the reliability of the test results of the questionnaires and found that Cronbach’s alpha equal to 0.985, indicating a high reliability for the results^86^.

### Color component index extraction

**Figure 3:**
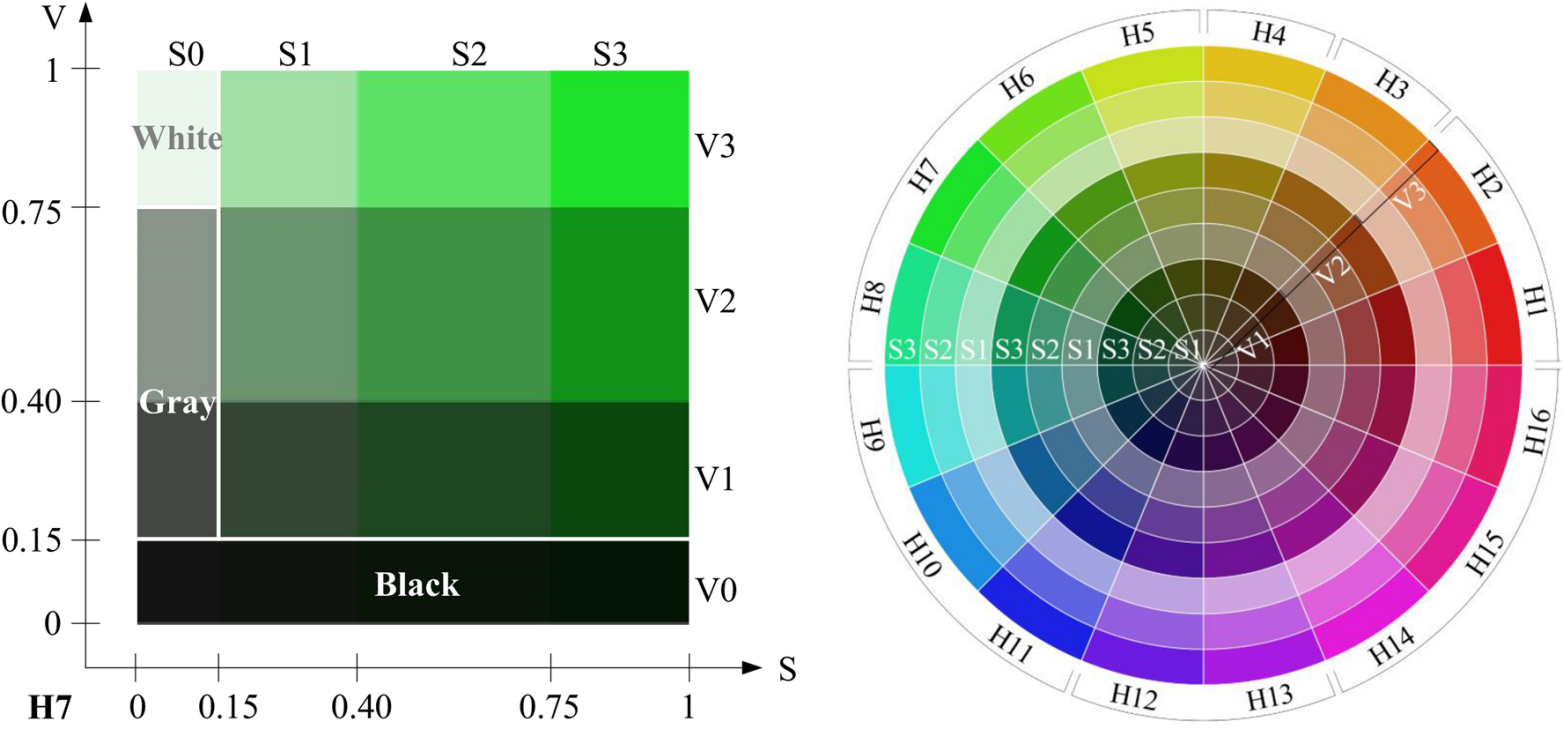
(**a**) Representative graph of 1 normalized (into black, white, and grays) color component index (H7) in the green hue category. S, saturation index; V, value index. (**b**) The 144 chromatic colors and the hue (H), saturation (S), and value (V) Indices used in our study. Adapted from Ref.^19^.

The HSV (hue, saturation, and value) model best describes both human color perception and psychophysiological responses to color, so it is widely used in studies of color perception and quantification^87,88^. Zhang et al.^19^ used the HSV color model to interpret color information in forest photos using a self-developed program on the MATLAB platform (MathWorks, Natick, MA, USA). That method extracts color components from a photo, including tiny amounts of color that are accurate to 1 pixel, and counts the proportion of different colors. We used that method to quantify color components using the following three steps: (1) All 120 digital photos (high-definition images of at least 4000 × 6000 pixels) were selected, and the sky elements were replaced with pure white backgrounds to facilitate a more accurate calculation of forest color components. (2) The HSV model was used to perform non-uniform quantification of the three attribute values (hue, saturation, and value) based on the color perception characteristics of the human eye^19^. (3) Because human eyes tend to simplify similar colors, they were normalized into black, white, and gray (Fig. 3a)^19,89^. Once the color components had been quantified, we had acquired the hue, saturation and value of 147 colors, including 144 chromatic colors (Fig. 3b) and three achromatic colors (i.e., black, white, and gray) (Fig. 3a), and automatically saved the ratio of each color in Microsoft Excel 2019 (Redmond, WA, USA). Next, we obtained 27 color component indices: (1) hue category ratio indices H1–H16, where H1 and H2, H3 and H4, H5–H9, H10 and H11, H12–H14, and H15 and H16 are members of the red, yellow, green, blue, blue-violet, and rose red series, respectively; (2) saturation ratio indices: S1 (low saturation), S2 (medium saturation), S3 (high saturation), gray, and white; (3) value ratio indices: V1 (low value), V2 (medium value), V3 (high value), and black; (4) number of colors (NC); and (5) maximum hue proportion index (MHI)^19^.

### Color patch index selection

**Figure 4:**
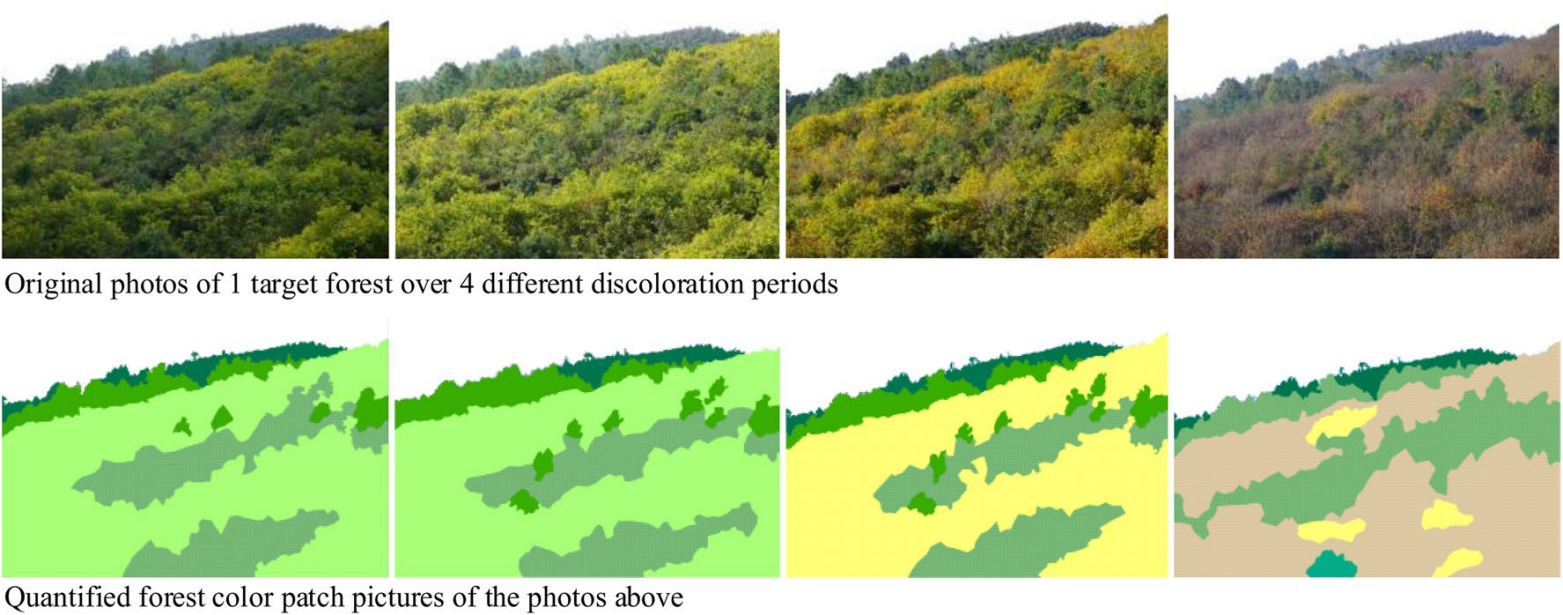
Color patch quantization.

The quantification of color component indices interpreted the color composition information of a photo, but the spatial attributes of the color distribution in the forest were still unknown. Li et al.^31^ showed that landscape indices that describe a landscape’s spatial structure characteristics accurately quantify forest color patch characteristics. Thus, using the relevant landscape indices and ArcGIS 10.2 (Environmental Systems Research Institute, Redlands, CA, USA), we used visual interpretation to draw out patches of different colors in each photo. The operation proceeded as follows: After we had fully magnified the pixels in our high-definition images, we used normal visual color discrimination to very accurately distinguish the color patches and outline their boundaries. Since coniferous forests, evergreen broad-leaf forests, deciduous broad-leaf forests, and shrub woods were mixed in some pictures, and their boundaries were indistinct, we classified the types of plants according to coniferous forests, broadleaf forests, and shrub woods, and placed plant species with similar colors in one patch (Fig. 4). We converted the SHAPE vector graphics into TIF raster images and imported them into Fragstats 4.2^90^ to calculate the color patch indices, ultimately selecting 20 indices (Table 1).

**Table 1:** Color patch indices.

Color patch indices	Abbreviations	Color patch indices	Abbreviations
Coniferous forest patch area proportion	AP	Perimeter areas fractal dimension of color patch	PAFRAC
Broad-leaved forest patch area proportion	BP	Contagion index of color patch	CON
Shrub wood patch area proportion	SP	Proportion of like adjacency of color patch	PLADJ
Grassland patch area proportion	GP	Interspersion juxtaposition index of color patch	IJI
Number of color patches	NP	Cohesion index of color patch	COH
Color patch density	PD	Division index of color patch	DIV
Largest color patch proportion index	LPI	Splitting index of color patch	SPLIT
Color landscape shape index	LSI	Shannon’s diversity index of color patch	SHDI
Mean color patch area	MPA	Simpson’s evenness index of color patch	SIEI
Color patch area coefficient of variation	ACOV	Aggregation index of color patch	AI

### Patch and color extraction of tree species

We outlined patches of every species of tree in the photos and interpreted the color change information of these tree species. Based on detailed identification of the tree species within the target slope forest areas, we fully enlarged each photo to the extent that features were clear enough for normal visual recognition in ArcGIS 10.2, and then patches of the same tree species were classified as a type of patch. For pictures with mixed plant species that were difficult to distinguish, we compared the color and shape of the same plants in different discoloration periods to determine the patch boundaries. Then, we intercepted the patches of each tree species in each discoloration period and imported them into the MATLAB 2016b to extract the color component indices (i.e., hue, value, and saturation).

### Data processing and statistical analyses

A one-way analysis of variance (ANOVA) and multiple comparison analyses were used to analyze the color indices and SBE values (dependent variables) in the various discoloration periods (independent variables). We then used a Pearson correlation analysis to analyze the correlations between the SBE values and color indices. The color indices that were significantly related to the SBE values were selected for factor analysis to construct color common factors^83^. Based on the correlations between the color indices and common factors, the common factor score coefficient was simplified to obtain simplified common factor equations. The correlations between the color common factors and the SBE values were then evaluated using Pearson’s correlation coefficients. Finally, the photos with the top 25% SBE values^91^, signifying the best visual aesthetic quality, were assigned through a systematic clustering method to categorical variables based on color common factors^19,92–94^. The significance level of all statistical analyses was set to *p* = 0.05. The data were analyzed using SPSS v. 25.0 (IBM, Inc, Armonk, NY, USA).

## Results

### Autumnal changes measured by slope forest color indices and SBE values

**Figure 5:**
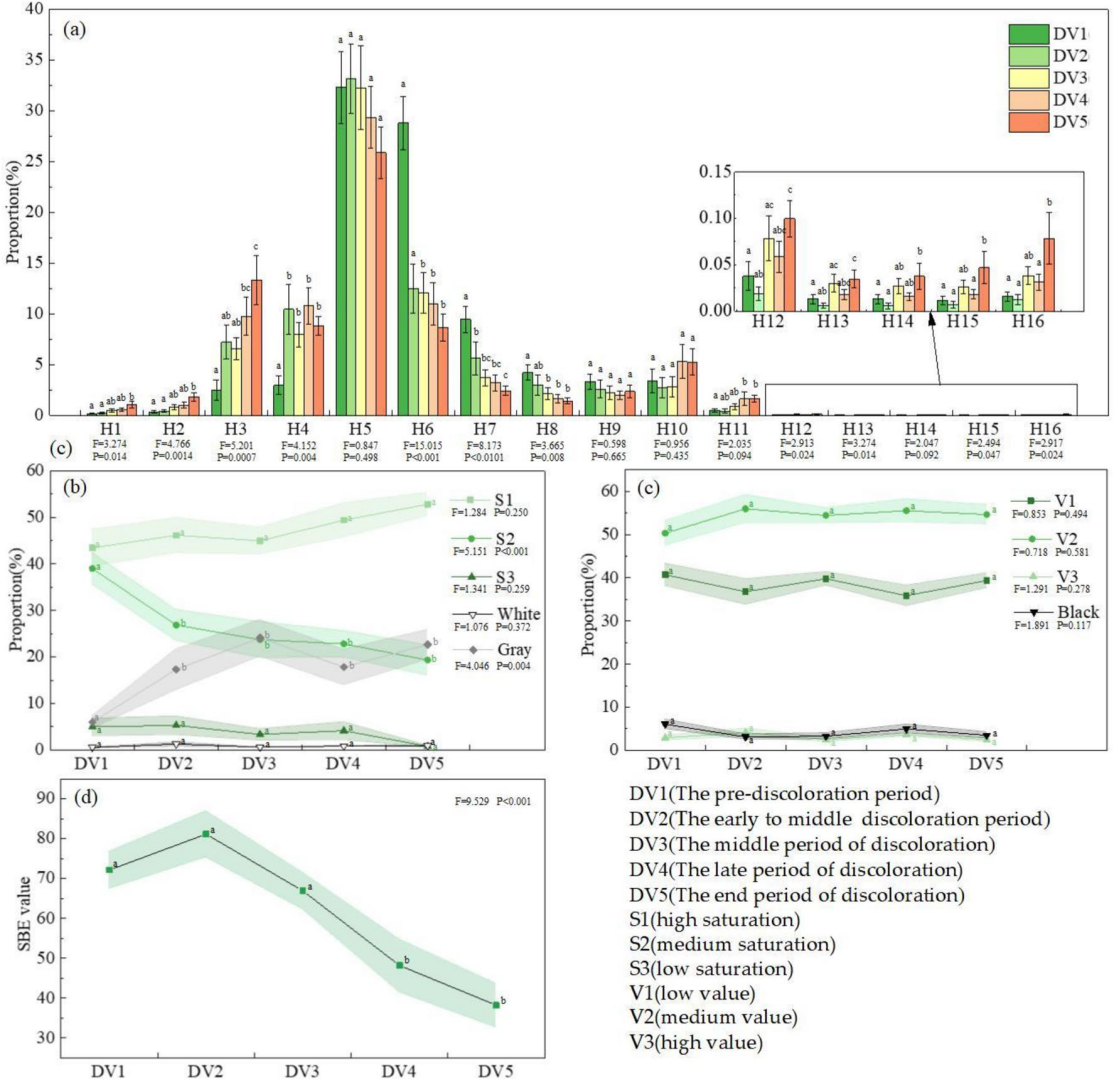
(**a**) Changes in hue (H) during the five autumn discoloration periods (DVS). The hue categories were H1–H2, red; H3–H4, yellow; H5–H9, green; H10–H11, blue; H12–H14, blue-violet; and H15–H16, rose red. Bars indicate the mean, and whiskers indicate SE. (**b–d**) Changes in saturation (**b**), value (**c**), and scenic beauty estimation (SBE) values (**d**) during the autumn discoloration periods. The shading along the lines indicates the SE. Different letters indicate significant differences at the 0.05 level.

Variations in the color component and patch indices for our study site progressed as follows (see Table S5 for more details). The proportion of the green hue series (H5, H6, H7, H8, H9) was the largest of all the hue series, but it gradually decreased over time, accompanied by different trends among the five hue categories (Fig. 5a). Proportionally, the yellow series (H3, H4) was second, and it gradually increased through time, while the blue (H10, H11) and red series (H1, H2) began relatively small but also gradually increased. The other color proportions (H12–H16) were always < 1%. In terms of saturation proportion, initially S1 (low saturation) > S2 (medium saturation) > S3 (high saturation), and the gray was lower than S2, while the white was slightly lower than S3 (Fig. 5b). During the discoloration periods, S2 decreased and gray increased, and both changes were significant (*p* < 0.05). S1 dominated in the autumn. The value index began with V2 (medium value) > V1 (low value) > V3 (high value) (Fig. 5c), and since black resembles V3, medium value colors occurred proportionally more than the others. As the autumn gradually advanced, the value index did not change significantly (*p* > 0.05). Over time, the number of colors (NC) fluctuated downward (18, 17.1053, 17.3478, 16.7857, and 17.2593, respectively) and the maximum hue proportion index (MHI) gradually decreased (40.2811, 35.0729, 33.6485, 33.6413, and 29.8917, respectively), but neither change was significant (*p* > 0.05). Finally, as autumn progressed, the color patch area coefficients of variation (ACOV) of the five discoloration periods (2.1942, 3.1021, 3.3728, 2.5936, and 2.5297, respectively) first increased and then decreased and in the middle period, reached a peak that was significantly greater than the values of other periods (*p* < 0.05). There were no significant changes (*p* > 0.05) in the other color patch indices.

The SBE values of the autumn forest on Jiaozi Mountain increased and then fell, so that the early to middle discoloration period scored the highest. The SBE values in the late and end discoloration periods were significantly lower than those of the first three discoloration periods (Fig. 5d, Table S5).

### Relationships between slope forest color indices and SBE values

**Figure 6:**
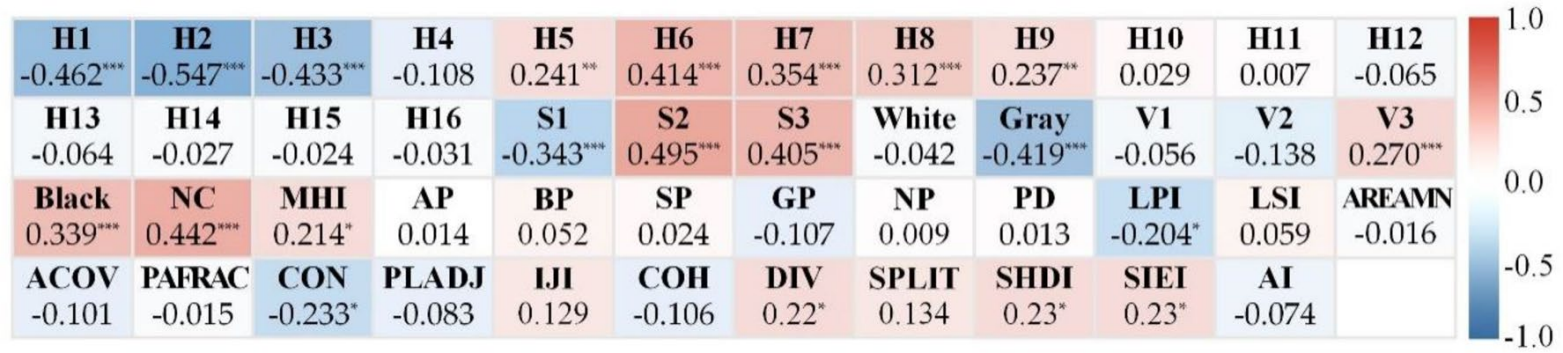
Results of the correlations between SBE values and color indices. *p < 0.05, **p < 0.01, ***p < 0.001. *NC* number of colors, *MHI* maximum hue proportion index; See Fig. 5 for definitions of H, S, and V and Table 1 for the remaining definitions.

The forest color indices that significantly positively correlated with SBE values had correlation coefficients that ranged (highest to lowest) from S2 > number of colors (NC) > H6 > S3 > H7 > black > H8 > V3 > H5 > H9 > Shannon’s diversity index of color patch (SHDI) > Simpson’s evenness index of color patch (SIEI) > Division index of color patch (DIV) > maximum hue proportion index (MHI). Those that were significantly negatively correlated had correlation coefficients that ranged in descending order from H2 > H1 > H3 > Gray > S1 > color patch contagion index (CON) > largest color patch proportion index (LPI) (Fig. 6). The rest of the color component indices and color patch indices did not significantly correlate with the SBE value, so they were eliminated from subsequent factor analysis.

As shown in Fig. 6, 21 color indices were significantly related to the SBE values, and there were overlaps and redundancies between those indices (Table S6). Therefore, to reduce the dimensionality of variables and produce more independence among the variables we used factor analysis to integrate those index variables with strong correlations into single common factors^83^. We found that the first six common factors (F1–F6) explained 85.925% of all the variables. Their simplified common factor Eqs. (4)–(9) are

4 $$F1=-0.224LPI-0.229CON+0.232DIV+0.19SHDI+0.24SIEI,$$

5 $$F2=0.185H6+0.286H7+0.268H8+0.192H9+0.22NC,$$

6 $$F3=0.265H5+0.21S2-0.343Grey+0.348MHI,$$

7 $$F4=0.352H1+0.389H2+0.315H3,$$

8 $$F5=-0.474S1+0.436Black,$$

9 $$F6=0.315S3+0.587V3.$$

**Figure 7:**
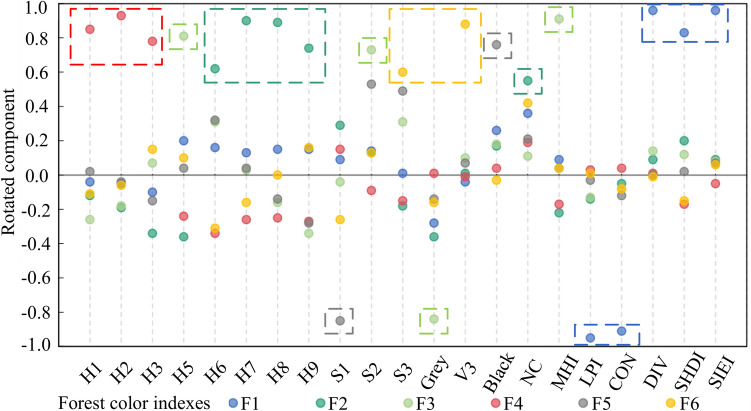
Rotated component matrix of the six common factors (F1–F6) produced using factor analysis of the 21 color indices that were significantly related to the scenic beauty estimation values. Each dashed box represents a common factor, and each holds those color indices that had significant correlations with that factor, as indicated by the factor color. See Fig. 6 for index definitions.

Figure 7 shows the 21 color indices rotated loading values in those six common factors, among which the variables in F1 primarily describe the integral pattern characteristics of color patch aggregation and dispersion. F1 became higher as the Shannon diversity index of color patch (SHDI), the Simpson’s evenness index of color patch (SIEI), and the Division index of color patch (DIV) increased, whereas the color patch contagion index (CON) and the largest color patch proportion index (LPI) decreased. This meant the color patches were scattered and had diverse equilibria. F2 was significantly related to H6, H7, H8, H9, and NC. F2 increased as the proportion of those hues and the number of colors increased. F3 primarily explained the maximum hue proportion index (MHI), H5, S2, and gray and larger F3 values had larger MHI and ratios of H5 and S2 but a smaller proportion of gray. F4 significantly correlated with H1, H2, and H3 and the F4 value grew larger as their proportions increased. F5 primarily described S1 and black and increased as the proportion of black became larger and the proportion of S1 became smaller. Finally, F6 was closely associated with S3, and V3 grew larger as their proportions increased.

We next tested the correlations of the six color common factors with the SBE values. The results showed that the SBE values were all significantly correlated (five positively and one negatively) with all six color common factors (F1, *r* = 0.222, *p* < 0.05; F2, *r* = 0.459, *p* < 0.01; F3, *r* = 0.385, *p* < 0.01; F4, *r* =  − 0.484, *p* < 0.01; F5, *r* = 0.379, *p* < 0.01; and F6, *r* = 0.403, *p* < 0.01). Thus, those color common factors were effective and reliable to be the categorical variables to classify photos with similar SBE values to obtain more detailed differences.

### Color combinations of forest photos of superior visual aesthetic quality

The forest photos that garnered the top 25% SBE values were considered superior visual aesthetic quality photos and divided into five categories (A, B, C, D, and E) based on six common color factors. Table 2 and Table S7 show the respective color index threshold ranges and the main color characteristics of those categories. The six color common factors were all significantly different among the five categories (*P*_A_ = 0.004; *P*_B_, *P*_C_, *P*_D_, and *P*_E_ < 0.001).

**Table 2:** Color combinations of the five superior visual aesthetic quality forest photo types (A–E).

Type	Color common factor differences	Main color characteristic interpretations	Example images
A		F1 and F2 were the highest among the five types. The color patch diversity was very high, and the proportion of the largest color patch was medium to low. The proportion of blue-green hue series was very high, and the ratios of the red and yellow series were very small. Saturation and value were at moderate levels	
B		Color patch diversity was relatively high. The red, yellow, and yellow-green hues (H5) were relatively more prominent, and the blue-green series accounted for a relatively small proportion among the five types (Table S6). Saturation and value were at medium levels	
C		The color patches were diverse and balanced. A high proportion of the hues were greens, and red and yellow colors were minimal. Saturation and value were at medium to high levels. Among the five types, F5 was at its highest here. Black was the highest, thus, leading to obvious value contrast (Table S6)	
D		Among the five types, F1 was at its lowest here. The color patches were highly agglomerated with the largest color patch comprising the largest proportion of the photo. The landscape structure was relatively simple. The proportion of low-value gray patches was very high, but there were high value, saturation, and red-yellow series in small patches scattered in the photo. They contrasted with the main gray patches	
E		Color patches were diversified, balanced, and had integral unity. Both the red and yellow series were proportionally high, while the blue-green series had a smaller proportion than A-C, and colors were relatively rich. Since F6 had the highest factor value, both the saturation and value of the overall photo were very high	

F1–F6, color common factors. Bars in the graphs indicate means, and whiskers indicate the SEs.

### Changes in tree species color in the slope forests with superior visual aesthetic quality

We screened the slope forests whose photos were most frequently assigned as having superior visual aesthetic quality and eventually selecting nine forests completely covered by trees species typical to the region. With 84 tree species identified (Table 3), we outlined the patch boundaries of each species in the nine superior forests and then calculated the color information, such as hue, value, and saturation, for each species in different discoloration periods. Finally, we summarized the 84 tree species and their color changes in Table 4.

**Table 3:** The 84 tree species identified were categorized according to their color dominance in the nine slope forests with superior visual aesthetic quality (forest type).

Forest type	Tree species composition		
	Primary tree species (40% < ratio ≤ 100%)	Adjunctive tree species (10% < ratio ≤ 40%)	Interspersed tree species (0% < ratio ≤ 10%)
1	*Castanea mollissima Blume (PC)*	*Pinus yunnanensis* Franch. (PC), *Alnus nepalensis* D. Don (PC), *Juglans sigillata* Dode (PC/AC)	*Pinus armandii* Franch. (IC), *Camellia reticulata* Lindl. (PC), *Quercus acutissima* Carr. (PC/AC), *Cyclobalanopsis glaucoides* Schotky (PC)
2	*Lithocarpus dealbatus* (J. D. Hooker et Thomson ex Miquel) Rehder (PC)	*Alnus nepalensis* D. Don *(PC), Pinus armandii* Franch. (PC), *Quercus spinosa David ex Franch. var.pseudosemicarpifolia* (PC/AC), *Pinus yunnanensis Franch.* (PC), *Quercus variabilis Blume* (AC)	*Cyclobalanopsis glaucoides* Schotky (PC), *Rhus chinensis* Mill. (PC/IC), *Toxicodendron succedaneum* (L.) O. Kuntze (IC/AC), *Quercus acutissima* Carr. (PC/AC/IC), *Lithocarpus mairei* (Schottky) Rehd (PC/AC), *Keteleeria evelyniana* Mast. (PC), *Lithocarpus leucostachyus* A. Camus (PC/IC), *Quercus aquifolioides* Rehd. et Wils. (PC), *Cyclobalanopsis augustinii* (Skan) Schottky (PC/AC)
3	*Castanopsis orthacantha* Franch. (PC)	*Lithocarpus mairei* (Schottky) Rehd (PC), *Quercus senescens* H. Léveillé (PC/AC)*, Pinus armandii* Franch. (PC/AC), *Cunninghamia lanceolata* (Lamb.) Hook. (PC), *Platycladus orientalis* (L.) Franco (PC/AC), *Alnus nepalensis* D. Don (PC/AC), *Populus davidiana* Dode (PC/AC)	*Lithocarpus leucostachyus* A. Camus (PC), *Alnus ferdinandi-coburgii* Schneid. (PC/IC), *Betula utilis* D. Don (PC/AC), *Platycarya strobilacea* Sieb. et Zucc. (PC/AC/IC), *Cyclobalanopsis glaucoides* Schotky (PC), *lithocarpus variolosus* (Franch.) Chun (PC/AC), *Litsea cubeba* (Lour.) Pers. (PC/AC), *Cornus walteri* Wangerin (PC), *Rhododendron decorum* Franch. (PC/AC), *Eurya handel-mazzettii* H. T. Chang (PC/AC), *Acer paxii* Franch. (PC/IC)
4	*Eurya loquaiana* Dunn (PC)	*Castanopsis orthacantha* Franch. (AC), *Ternstroemia gymnanthera* (Wight et Arn.) Beddome (PC/AC), *Dryopteris wallichiana* (Spreng.) Hylander (PC/AC)	*Pinus armandii* Franch. (PC), *Symplocos theaefolia* D. Don (PC), *Illicium simonsii* Maxim. (PC), *Populus davidiana* Dode (PC/IC), *Acer paxii* Franch. (PC/IC), *Acer davidii* Franch. (PC/IC), *Meliosma cuneifolia* Franch. *var. glabriuscula* Cufod. (PC/AC), *Rhododendron virgatum* Hook. f. (PC), *Acer sinense* Pax (PC/IC), *Camellia pitardii* Cohen-Stuart (PC), *Lithocarpus mairei* (Schottky) Rehd (PC), *Schima argentea* Pritz. ex Diels (PC/IC), *Quercus aquifolioides* Rehd. et Wils. (PC), *Phoebe forrestii* W. W. Smith. (PC), *Toona ciliate* Roem. (PC), *Quercus spinosa* David ex Franchet var. *spinosa* (PC), *Salix wallichiana* Anderss. (PC/IC)
5	*Cyclobalanopsis glaucoides* Schotky (PC), *Lithocarpus mairei* (Schottky) Rehd (PC)	*Alnus nepalensis* D. Don (PC/AC), *Pinus yunnanensis* Franch. (PC/AC), *Berberis cavaleriei* Lévl. (PC/AC), *Lonicera acuminata* Wall. (PC/AC)	*Pinus armandii* Franch. (IC), *Juniperus squamata* Buchanan-Hamilton ex D. Don (PC), *Litsea pungens* Hemsl. (PC), *Populus adenopoda* Maxim. (PC/IC), *Populus davidiana* Dode (PC/IC), *Acer oliverianum* Pax (PC/IC), *Acer cappadocicum* Gled. subsp. *sinicum* (Rehder) Handel-Mazzetti (PC/IC), *Acer davidii* Franch. (PC/IC), *Quercus rehderiana* Handel-Mazzetti (PC/IC), *Quercus guyavifolia* H. Léveillé (PC/IC), *lithocarpus variolosus* (Franch.) Chun (PC)
6	*Pinus densata* Mast. (PC)	*Populus davidiana* Dode (AC), *Pinus armandii* Franch. (AC), *Alnus nepalensis* D. Don (PC/AC), *lithocarpus variolosus* (Franch.) Chun (PC/AC)	*Pinus yunnanensis* Franch. (PC/AC/IC), *Quercus spinosa* David ex Franch. var. *pseudosemicarpifolia* (PC/AC)
7	*Salix praticola* Hand.Mazz. ex Enand. (PC)	*lithocarpus variolosus* (Franch.) Chun (PC/AC), *Acer laxiflorum* Pax (PC/AC), *Salix wallichiana* Anderss. (PC/AC)	*Quercus aquifolioides* Rehd. et Wils. (PC), *Sorbus coronate* (Card.) Yü et Tsai (PC/IC), *Acer sterculiaceum* Wall. subsp. *franchetii* (Pax) A. E. Murray (PC/AC), *Acer sterculiaceum* Wall. (PC/AC), *Acer caudatum* Wall var. *prattii* Rehd (PC/AC), *Litsea chunii* Cheng (PC/AC), *Betula utilis* D. Don (PC/AC), *Quercus guyavifolia* H. Léveillé (PC), *Quercus spinosa* David ex Franch. var. *pseudosemicarpifolia* (PC), *Populus davidiana* Dode (PC/AC), *Populus rotundifolia* griff. var. *duclouxiana* (Dode) Gomb. (PC/AC)
8	*Acer flabellatum* Rehd. (PC)	*Rhododendron fastigiatum* Franch. (PC), *Acer forrestii* Diels (PC/AC), *Litsea pungens* Hemsl. (PC/AC)	*Acer caudatum* Wall.var. *prattii* Rehd (PC/AC), *Populus rotundifolia* griff. var. *duclouxiana* (Dode) Gomb. (PC), *Salix brachista* Schneid. (PC/AC/IC), *Acer laxiflorum* Pax (PC/AC/IC), *Betula potaninii* Batal. (PC/AC/IC), *Sorbus multijuga* Koehne (PC /IC), *Quercus guyavifolia* H. Léveillé (PC), *Juniperus squamata* Buchanan-Hamilton ex D. Don (IC), *Rhododendron alutaceum* Balf. F. et W. W. Smith (PC)
9	*Rhododendron delavayi* Franch. (PC)	*Litsea pungens* Hemsl. (PC/AC), *Rhododendron decorum* Franch. (PC/AC), *Rhododendron sphaeroblastum* Balf. F. (PC), *Rhododendron irroratum* Franch. (PC), *Rhododendron racemosum* Franch. (PC)	*Berberis woomungensis* C. Y. Wu ex S. Y. Bao (PC/ IC), *Rhododendron complexum* Balf. F. et W. W. Smith (PC), *Sorbus rufopilosa* Schneid. (PC/IC), *Sorbus rehderiana* Koehne var. *rehderiana* (PC/AC), *Hydrangea heteromalla* D. Don (PC), *Rhododendron lacteum* Franch. (PC/AC), *Acer caudatum* Wall.var. *prattii* Rehd (PC/IC), *Yushani a maculate* T. P. Yi (PC), *Rhododendron alutaceum* Balf. F. et W. W. Smith (PC), *Rhododendron fastigiatum* Franch. (PC)

*PC* primary color, *AC* adjunctive color, *IC* interspersed color.

**Table 4:** Classification of the characteristics of color change of tree species.

Color change type	Primary tree species (40% < ratio ≤ 100%)	Adjunctive tree species (10% < ratio ≤ 40%)	Interspersed tree species (0% < ratio ≤ 10%)
**Evergreen**			
Mainly green (H6, H7, H8)	*Cyclobalanopsis glaucoides* Schotky (PC), *Eurya loquaiana* Dunn (PC), *Rhododendron delavayi* Franch. (PC)	*Platycladus orientalis* (L.) Franco (PC/AC), *Ternstroemia gymnanthera* (Wight et Arn.) Beddome (PC/AC), *Rhododendron sphaeroblastum* Balf. F. (PC), *Rhododendron irroratum* Franch. (PC), *Rhododendron decorum* Franch. (PC/AC), *Rhododendron racemosum* Franch. (PC)	*Camellia reticulata* Lindl. (PC/AC), *Cyclobalanopsis augustinii* (Skan) Schottky (PC/AC)*, Rhododendron decorum* Franch. (PC/AC), *Eurya handel-mazzettii* H. T. Chang (PC/AC), *Symplocos theaefolia* D. Don (PC), *Rhododendron virgatum* Hook. f. (PC), *Illicium simonsii* Maxim. (PC), *Camellia pitardii* Cohen-Stuart (PC), *Toona ciliate* Roem. (PC), *Juniperus squamata* Buchanan-Hamilton ex D. Don (PC/IC)
Yellow-green (H5, H6)	*Pinus densata* Mast. (PC)	*Rhododendron fastigiatum* Franch. (PC), *Quercus spinosa* David ex Franch. var. *pseudosemicarpifolia* (PC/AC), *Pinus armandii* Franch. (PC/AC)	*Quercus rehderiana* Handel-Mazzetti (PC/IC), *Quercus guyavifolia* H. Léveillé (PC/IC), *Quercus spinosa* David ex Franch. var. *pseudosemicarpifolia* (PC/AC), *Rhododendron alutaceum* Balf. F. et W. W. Smith (PC), *Rhododendron fastigiatum* Franch. (PC), *Rhododendron lacteum* Franch. (PC/AC), *Rhododendron complexum* Balf. F. et W. W. Smith (PC), *Yushania maculate* T. P. Yi (PC)
Green (H6, H7, H8) to yellow-green (H5, H6)	*Lithocarpus dealbatus* (J. D. Hooker et Thomson ex Miquel) Rehder (PC)	*Berberis cavaleriei* Lévl. (PC/AC), *Lonicera acuminata* Wall. (PC/AC), *Pinus armandii* Franch. (PC/AC)	*Lithocarpus leucostachyus* A. Camus (PC/IC), *Cyclobalanopsis glaucoides* Schotky (PC)
Blue-green (H7, H8, H9)	*Pinus armandii* Franch. (PC), *Lithocarpus mairei* (Schottky) Rehd (PC), *Castanopsis orthacantha* Franch. (PC)	*Quercus senescens* H. Léveillé (PC/AC), *Lithocarpus mairei* (Schottky) Rehd (PC), *Pinus armandii* Franch. (PC/AC), *Cunninghamia lanceolata* (Lamb.) Hook. (PC), *lithocarpus variolosus* (Franch.) Chun (PC/AC), *Castanopsis orthacantha* Franch. (AC)	*Pinus armandii* Franch. (PC/IC), *Keteleeria evelyniana* Mast. (PC), *Lithocarpus leucostachyus* A. Camus (PC/IC), *lithocarpus variolosus* (Franch.) Chun (PC/AC), *Lithocarpus mairei* (Schottky) Rehd (PC/AC), *Phoebe forrestii* W. W. Smith. (PC), *Quercus spinosa* David ex Franchet var.*spinosa* (PC)
Green (H6, H7, H8) to yellow (H3, H4) and green (H5, H6, H7) variegation		*Pinus yunnanensis* Franch. (PC/AC)	*Schima argentea* Pritz. ex Diels (PC/IC), *Pinus yunnanensis* Franch. (PC/AC/IC)
**Deciduous**			
Green (H5, H6, H7, H8) to withered yellow (H3, H4) and withered red (H1, H2) and gray		*Alnus nepalensis* D. Don (PC/AC), *Dryopteris wallichiana* (Spreng.) Hylander (PC/AC)	*Alnus ferdinandi-coburgii* Schneid. (PC/IC), *Cornus walteri* Wangerin (PC), *Berberis woomungensis* C. Y. Wu ex S. Y. Bao (PC/ IC), *Hydrangea heteromalla* D. Don (PC), *Quercus aquifolioides* Rehd. et Wils. (PC)
Green (H5, H6, H7, H8) to yellow (H4) to withered yellow (H3, H4) and gray	*Salix praticola* Hand Mazz. ex Enand. (PC), *Acer flabellatum* Rehd. (PC)	*Quercus variabilis* Blume (AC), *Juglans sigillata* Dode (PC/AC), *Populus davidiana* Dode (PC/AC), *Acer laxiflorum* Pax (PC/AC), *Salix wallichiana* Anderss. (PC/AC), *Litsea pungens* Hemsl. (PC/AC)	*Betula utilis* D. Don (PC/AC), *Platycarya strobilacea* Sieb. et Zucc. (PC/AC/IC), *Litsea cubeba* (Lour.) Pers. (PC/AC), *Quercus acutissima* Carr. (PC/AC/IC), *Acer paxii* Franch. (PC/IC), *Populus davidiana* Dode (PC/AC/IC), *Meliosma cuneifolia* Franch. *var. glabriuscula* Cufod. (PC/AC), *Acer davidii* Franch. (PC/IC), *Acer sinense* Pax (PC/IC), *Salix wallichiana* Anderss. (PC/IC), *Litsea pungens* Hemsl. (PC), *Populus adenopoda* Maxim. (PC/IC), *Acer oliverianum* Pax (PC/IC), *Acer cappadocicum* Gled. subsp. *sinicum* (Rehder) Handel-Mazzetti (PC/IC), *Acer sterculiaceum* Wall. subsp. *franchetii* (Pax) A. E. Murray (PC/AC), *Acer sterculiaceum* Wall. (PC/AC), *Acer caudatum* Wall var. *prattii* Rehd (PC/AC/IC), *Litsea chunii* Cheng (PC/AC), *Populus rotundifolia* griff. var. *duclouxiana* (Dode) Gomb. (PC/AC), *Salix brachista* Schneid. (PC/AC/IC), *Acer laxiflorum* Pax (PC/AC/IC), *Betula potaninii* Batal. (PC/AC/IC)
Green to yellow (H4) to yellow (H3) to withered yellow (H3, H4) and gray	*Castanea mollissima* Blume (PC)	*Acer forrestii* Diels (PC/AC)	*Rhus chinensis* Mill. (PC/IC)
Green to yellow (H3, H4) to red (H1, H2) to withered red (H1, H2) and gray			*Toxicodendron succedaneum* (L.) O. Kuntze (IC/AC), *Sorbus coronate* (Card.) Yü et Tsai (PC/IC)
Green to red (H1, H2) to withered red (H1, H2) and gray			*Sorbus multijuga* Koehne (PC/IC), *Sorbus rufopilosa* Schneid. (PC/IC), *Sorbus rehderiana* Koehne var. *rehderiana* (PC/AC)

*PC* primary color, *AC* adjunctive color, *IC* interspersed color.

**Figure 8:**
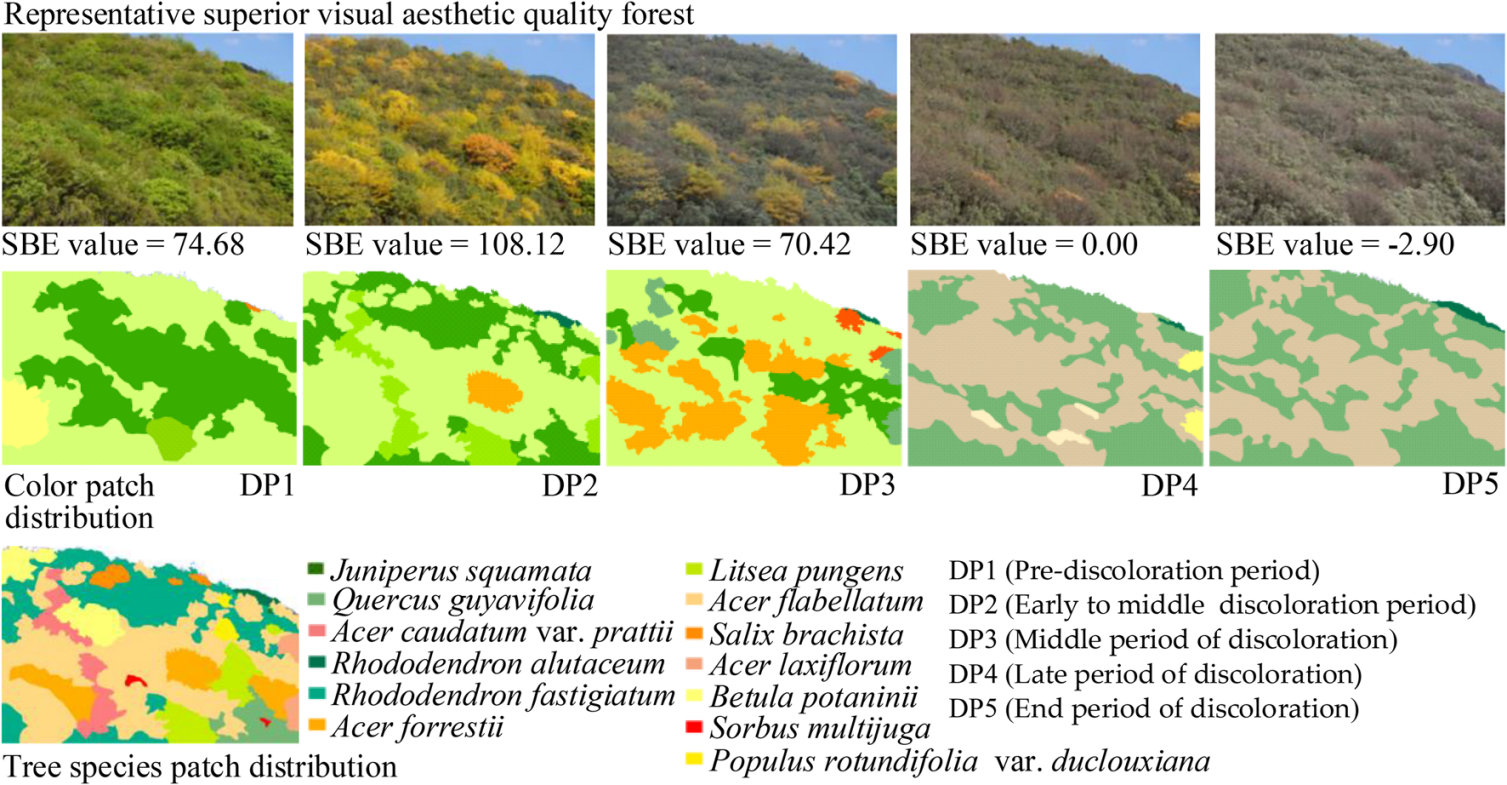
Overlay analysis of tree species patch distributions and color patch distributions in one representative superior slope forest through the autumn discoloration period. *SBE* scenic beauty estimation.

To distinguish the dominance of each of those tree species in the overall forest, we calculated the areal ratio of each species in the forest (Fig. 8) and assigned them into one of three categories based on those ratios: primary tree species (40% < ratio ≤ 100%), adjunctive tree species (10% < ratio ≤ 40%), and interspersed tree species (0% < ratio ≤ 10%)^95^. We used that same method to distinguish the degree of dominance of each color in the overall forest color palette, classifying the patch distributions of forest color in different autumnal discoloration periods as primary color (40% < ratio ≤ 100%), adjunctive color (10% < ratio ≤ 40%), and interspersed color (0% < ratio ≤ 10%)^95^. We then performed an overlay analysis to determine which dominant color types were presented by each species in different autumnal discoloration periods (Fig. 8). The dominant color type of each species was divided into a primary color, adjunctive color, and interspersed color (Table 3). Finally, we identified 16 primary, 23 adjunctive, and 62 interspersed tree species. If the same tree species repeatedly presented the same color dominance types in different forests, they were counted only once. Thus, the primary tree species also presented primary color dominance (e.g., *Castanea mollissima* Blume); adjunctive tree species presented primary or adjunctive color dominance, or both, depending on the autumnal discoloration period (e.g., *Juglans sigillata* Dode), and the interspersed tree species presented one to three color dominances, also depending on the autumnal discoloration period (e.g., *Quercus acutissima* Carr*.*).

The changes in autumn color between evergreen and deciduous tree species differed markedly (Table 4). Evergreens (43 species) were generally green with partial transitions to yellow or blue, including five main types of color changes. The changes in color of the deciduous trees (41 species) were more obvious than those of the evergreens, with richer color types of green, yellow, red, withered yellow, withered red, and gray. In addition, there were five main types of color changes, most of which were trees (26 species) that changed from green (H5, H6, H7, and H8) to yellow (H4) to withered yellow (H3, H4) and then gray.

Evergreens usually showed medium–low saturation that decreased slightly as autumn advanced, and their values were always maintained at a medium–low level with few apparent changes. When the deciduous tree species were green, red, and yellow, their saturation and value were higher than those of the evergreen tree species during the same discoloration period. However, as the discoloration period advanced, they primarily became withered yellow, withered red, and gray, and the saturation decreased significantly, becoming lower than that of the evergreen tree species. In addition, the value of deciduous trees decreased as the discoloration period advanced (Fig. 8).

## Discussion

### Slope forest color index and SBE value changes in the autumn

The color composition of Jiaozi Mountain forests obviously varied throughout the autumn, while changes in the distributions of color space were not so apparent. In particular, we found that the hues changed from the dominant green series to diverse colors (red, yellow, blue, green, and gray). This result complemented previous research^22,34,96,97^, which indicates that forest autumn colors change from green to red, yellow, and orange as determined using a precise color quantification method. The initial medium to low saturation dominance gradually decreased with the progression of color variation, but the variation of the value index was not significant and remained at mostly medium to low throughout the season. Neither the number of colors (NC) nor the maximum hue proportion index (MHI) changed significantly. These results were similar to those of Zhang et al.^19^ for saturation, but not for value, NC, and MHI. Their results showed that value and NC increased significantly, and MHI decreased significantly in the autumn. Those differences from our study may be owing to the differences in vegetation types and discoloration phenology periods of both study areas. Except for the color patch area coefficients of variation (ACOV), the other color patch indices lacked significant changes. This could be because the composition of forest color patch depends primarily on the distribution of the tree species, which were stable in the autumn^94,96,98^. The initial increase and then decline in the SBE values as the autumn discoloration progressed was probably caused by the changing forest colors^31,34,99^.

### Relationships between slop forest color and SBE value

The relationships between color components and SBE values were primarily divided into four attributes: hue, saturation, value, and color richness. Most studies^20,34,100,101^ have shown that people prefer green, red, and yellow hues, and our results support and contribute to those previous findings. While we found that the green series (H5, H6, H7, H8, H9) positively correlated with the SBE values, and the red (H1, H2) and yellow (H3, H4) color series were negatively related to the SBE values overall (Fig. 6). However, red and yellow were indeed related to high SBE values in categories B, D, and E (Table 2). In addition, we noted that as the color change progressed, some of the deciduous tree species turned yellow and red before turning withered red and withered yellow (e.g., *Acer davidii* Franch.). However, some trees (e.g., *Alnus nepalensis* D. Don) immediately changed from green straight to withered yellow and withered red, which resulted in more withered yellow and withered red than red and yellow for overall color ratio. Therefore, most likely red, yellow, and green positively correlated, while withered red and withered yellow negatively correlated with the SBE values. These results probably arose from the belief that green represents vitality and comfort; red and yellow suggest warmth and ripened fruit, while withered red and withered yellow may represent nutrient deficiencies, dormancy, or senescence^97,102^. These related emotions and entities influence the aesthetic preference for the color of trees^99,103^. Additionally, we showed that an increase in gray reduced the SBE value, but Kendal et al.^61^ claimed that humans are fond of gray. Perhaps their results were because their evaluators were gardeners who, unlike the general public, can be aesthetically tired of the abundant plant colors they face in their routine work and perhaps prefer a break that includes soothing colors with low saturation, such as gray. The evaluators in our study were drawn from the general public and they preferred red, yellow, or green plant colors to gray, since most of the artificial infrastructure, such as roads, that they face every day are primarily gray, which probably appear boring and tiring^20^. In addition, other socio-demographic characteristics, such as cultural groups, sex, and age, may also affect the evaluation of visual aesthetic preferences^47,99,104^. However, this study did not focus on these variables. Future research is necessary to investigate social demographic indices to better understand the variation in the influence of forest color on the visual aesthetic perception.

The positive relationships of saturation and value with SBE values are consistent with previous studies^19,93^. Our study supports these results. We found that high saturation (S3), medium saturation (S2) and high value (V3) had a significantly positive relationship with SBE, and gray had a significantly negative relationship with SBE. However, we also found that some superior forests had a small amount of black (the lowest saturation and value) with high value colors, which together formed a light and dark contrast (e.g., C in Table 2). A few superior forests were dominated by gray (the lowest saturation, lower value) patches interspersed with several small bright red and yellow patches, which together possess strong saturation and value contrast (e.g., D in Table 2). The SBE values of those forests were also high, thus, indicating that lower saturation and value, along with obvious high-low contrast, could also represent greater beauty^21^. This result is consistent with those of previous studies^105–107^, which concluded that the color contrast has a substantial influence on visual aesthetics^108^, and the subjects preferred landscapes with a higher variance in color^20,21^.

Previous studies have suggested that color diversity has a positive effect on visual aesthetics^20,21,36,39,48,93,97,106,109^. This study partially supports this result and showed that both the number of colors (NC) (r = 0.442, p < 0.001) and the maximum hue proportion index (MHI) (r = 0.214, p < 0.05) were positively correlated with the SBE values. This indicates that rich colors can indeed increase the degree of beauty, but a certain proportion of the main hues were needed to unify the overall color and to coordinate primary and secondary contrasts, which agrees with the relevant aesthetic principles^110^. Ma et al.^20^ also indicated that there is a threshold for the number of colors of the superior aesthetic landscape, and an excessive visual stimulus entailed negative effects^111^. But what two threshold values would maximize the benefits of balancing the number of colors and the maximum hue proportion index? This needs to be determined by a large number of repetitive measurements in different types of slope forests.

Among the color patches, spatial structures were relatively complex and featured both diversity and equilibrium, and largest color patch proportion index (LPI) was moderately low, thus, yielding greater SBE values. Zhang et al.^19^ had similar results for their out-of-forest landscapes, Mao et al.^94^ for their in-forest landscapes and Ma et al. for their community landscapes^20^. However, we also had higher SBE values assigned to photos when their largest color patch occupied a substantial proportion of the photo, but there were many smaller, interspersed color patches with color indices, such as the hue, saturation, and value, that contrasted with that largest one (e.g., D in Table 2). The result added to previous studies^19,20,31,94^.

### Different superior visual aesthetic forest photos vary in their color combinations

Few studies have explored the combination relationship of forest color indices, although the combination effect is very important for the optimization of slope forest landscapes^20,112^. We found that the color composition indices and color patch indices jointly affect the overall visual aesthetic. For example, Table 2D has a small number of colors and a high degree of color patch aggregation, while Table 2E has a large number of colors and rich color patches. Although they have different color combination characteristics, they both show that integral forest colors have a high visual aesthetic quality. This may be because a certain color patch has both color component attribute and patch shape attribute. This indicates that comprehensive and appropriate control of the color characteristics index threshold ranges and combinations is the key to obtaining forest colors with superior aesthetic qualities. Therefore, forest color optimization should not only consider the threshold range of a single color index but also consider its interaction with other indicators. The achievement of this goal first requires configuring those specific tree species colors with colors composed of superior forest colors.

### Tree species color changes in the superior visual aesthetic quality forests

Among the 84 tree species from the nine superior visual aesthetic quality forests, the changes in autumn color of the evergreens remained mostly within the green series. Compared with the evergreens, the color changes of deciduous tree species were more obvious and diverse, changing from green, to yellow and red, which possess higher saturation and value, and proceeding to withered yellow, withered red, and gray, which have lower saturation and value. Moreover, the SBE value first increased and then decreased (Fig. 8). This result is consistent with previous research^20,48^, which shows that deciduous trees have a higher aesthetic quality than evergreen trees when their foliage is discolored, such as red or yellow, in the autumn and that foliated scenes are preferred to dormant and senescent scenes^97,113^. Additionally, evergreen trees are primarily members of the Pinaceae, Fagaceae, Theaceae and Rhododendron, while deciduous trees are primarily members of the Salicaceae, Aceraceae and Betulaceae. This is consistent with previous studies on zonal vegetation types in southwest China ^31,70,114^. This indicates that the combination of these native tree resources not only has visual aesthetic advantages but also has the potential to maintain local ecological sustainability and native biodiversity restoration; in addition, it has the potential for large-scale planting in the slope forests that surround local cities^10,17,18^. Further, researching the ecological suitability and color variations of the tree species of this study in different regions is worth to be done in the future study.

### Strategies for the optimization of slope forest color

Our findings of the relationships between the variations in slope forest autumn color and the corresponding visual aesthetic quality enabled us to classify the color combination features of slope forests that display superior visual aesthetic quality. In addition, we were able to define the tree species compositions in forests with superior visual aesthetic quality and the color changes of each tree species. This led us to propose strategies for future forest color optimization by selecting, substituting, and supplementing those tree species that possess the color components that optimize the composition of color patches in the slope forest.

First, since the forests that need to be optimized may have low SBE values during one (or more) discoloration periods, the target primary hue type for those periods, such as green-dominated, yellow-dominated, green-yellow with 2-color contrast, red-yellow with 2-color contrast, and red-yellow-green with 3-color contrast, should be determined. Secondly, tree species should be selected based on their ecological dominance and the types of color change listed in Tables 3 and 4. When selecting tree species, we can first determine the primary color and primary tree species based on the target hue type determined and then determine the adjunctive color, adjunctive tree species, interspersed color, and interspersed tree species in turn^95^. Third, some of the existing plants in the forests that need to be optimized may be replaced, or those forests may be supplemented with the selected tree species^20,49^. The different periods of phenological color of various tree species should be considered^19,31,34,35^. Tree species with the same or different colors may exist together in the same discoloration period and also appear staggered during the asynchronous discoloration period. Species should be used together to form superior visual aesthetic quality colors for the integral forest during different discoloration periods. For example, for primarily deciduous tree forests (Fig. 8), the beauty of the forest decreases after the deciduous trees lose their foliage. Therefore, deciduous tree species with different phenological discoloration periods can be configured in equilibrium, so that discoloration is staggered to extend the duration of preferred colors, such as green, red, and yellow. In addition, evergreen and deciduous tree species may be configured in a balanced layout to reduce the impact of withered yellow, withered red, and gray after deciduous tree defoliation. Fourth, while adjusting the hue type, the saturation, value, number of colors (NC) and maximum hue proportion index (MHI) should also be considered to produce desirable hue, saturation, value, color richness, and contrasting relationships. Controlling those color component indices can be accomplished by referring to Table 2 and Supplementary Table S7 for the threshold ranges and combination relationships. Furthermore, since we found that both the species and number of red tree species in the Jiaozi Mountain forests in autumn are very small, their numbers should be increased in suitable habitats^19,20^.

The threshold ranges and combination relationships of the color patch indices in superior forests in Table 2 and Supplementary Table S7 should also be consulted, so that the integral color patch space leads to a diverse and balanced effect. If an area that requires optimization has enough superior color tree species for one (or more) discoloration periods, but their spatial configuration is poor, we should ensure that the original numbers of tree species and proportions remain basically unchanged as the spatial composition relationship is adjusted^20,31,49^. However, if there is a shortage of superior color tree species for one (or more) discoloration periods in an area requiring optimization, and the spatial configuration is poor, the tree species that both have superior color during those discoloration periods and can adapt to the conditions of that habitats should be selected and substituted in key positions^31,49^.

Indeed, the strategies describe above require continuous readjustment and improvement to verify their scientific value and aesthetic effectiveness. More comprehensive steps should also be considered for actual forest color optimization, and the main objectives of each situation should be clarified. For example, does a single view within the main target improve over repeated aesthetic experiences over time, or should the aesthetic experience of many different scenes during a single tour (at one time) be the focus of optimization? In addition, how should both these goals be balanced while formulating the most suitable optimization plan?

Additionally, owing to the long growth cycle of tree species, the actual process may be affected by other inevitable factors^49^. Thus, it is necessary to conduct more research to simulate forest color spatiotemporal variation based on accurate physiological and color information of each tree species. This can accurately and comprehensively evaluate and predict the visual aesthetic value of forest color and implement the specific tree species configuration.

### Limitations

The main limitation of this study was some inaccuracy in the identification of tree species because the long distance between observers and trees made it difficult to always ensure the identification of precise plant species. However, we used a combination of methods, including on-site visual recognition and identification based on photos of the plant and plant specimens described in the “Materials and methods” section, to strive for accurate tree species identification. Moreover, because the colors and ecological adaptation ranges of related tree species (within the same family or genus) were somewhat similar, those related species could be substituted for each other in actual configuration and use^114^. As a result, our final interpretation of tree species had adequate reliability and guidance. Additionally, forest selection could be subjective^34,115^, and the lack of real-time tree color variation monitoring may have led to missed information^30^. In addition, there may be some deviations when humans distinguish and outline color patches. Except for the color indices that were significantly linearly related to the SBE values in this article, other indices may have had non-linear relationships with the SBE values, and they require further exploration^19^. Finally, the lack of comprehensive comparisons of forest color variations during all four seasons and over many years may limit the applicability of the results^112^.

## Conclusions

This study is the first to apply the results of visual aesthetic evaluation of color changes in autumn slope forests to the configurations of corresponding tree species. A natural slope forest area was selected as the research object to provide visual aesthetic law, ecological sustainability and support for native biodiversity for urban slope forest management. The results indicated the following that led to the acceptance of all of the hypotheses: (1) The visual aesthetic quality first increased and then decreased as the natural slope forest color varied in the autumn. (2) The hallmarks of higher visual aesthetic quality included more green, red, and yellow; moderately higher saturation and value; more obvious color contrast; diverse colors; coordinated primary and secondary contrasts, diverse and balanced color patches; and a dominant color patch contrasted by many small patches with interspersed color components. (3) Different color combination features can achieve high visual aesthetic quality. (4) Most of the 84 tree species we found in the nine forests that had superior visual aesthetic quality displayed one of 10 types of color changes, and the evergreen and deciduous tree species had five types each. They primarily varied in green, yellow, blue, red, withered yellow, withered red and gray. Based on our results, we proposed operable strategies, including the selection, substitution, and supplementation of tree species, for slope forest color optimization.

## Supplementary Information

Supplementary Tables.
