# Supplementary material for: Implementation of the visual aesthetic quality of slope forest autumn color change into the configuration of tree species
Source: Sci Rep. 2022 Jan 20;12:1034. doi: 10.1038/s41598-021-04317-1 (PMC8776743; doi:10.1038/s41598-021-04317-1)
Supplement: Supplementary file 1 — Supplementary Tables. [file 41598_2021_4317_MOESM1_ESM.docx]

**Implementation of the visual aesthetic quality of slope forest autumn color change into the configuration of tree species**

**Yanxia Mu, Wenyue Lin, Xiuli Diao, Zhe Zhang, Jin Wang, Zijing Lu, Wencheng Guo, Yu Wang, Chunxiang Hu, Changyou Zhao**

**Table S1.** Vertical climate type characteristics of Jiaozi Mountain National Natural Reserve^1^.

| **Altitude (m)** | **Climate Type** | **Annual Mean Temperature (°C)** | **Mean Temperature of the Coldest Month (°C)** | **Mean Temperature of the Warmest Month (°C)** | **≥10℃ Accumulated Temperature (°C)** | **Annual Precipitation (mm)** | **Major Climatic Characteristics** |
| --- | --- | --- | --- | --- | --- | --- | --- |
| 1100-1400 | Southern subtropical climate of the valley | ＞19.6 | 12.4 | ≥24.3 | ≥6000 | 850.0 | Semi-humid monsoon climate, less precipitation and large evaporation |
| 1400-1600 | Central subtropical climate of the valley | 15.0-18.0 | 8.0-10.0 | 21.0-23.0 | 5200-6000 | 800.0-950.0 | Semi-humid monsoon climate, with little precipitation, large evaporation, and low air humidity |
| 1600-2000 | Northern subtropical climate of the mountain | 13.0-15.0 | 6.0-8.0 | 18.0-20.0 | 4800-5200 | 950-1100 | Humid monsoon climate of the plateau with abundant precipitation, high air humidity and slightly lower heat |
| 2000-2400 | Warm temperate climate of the mountain | 11.0-13.0 | 4.0-6.0 | 16.0-18.0 | 4200-4800 | 1100-1200 | Humid monsoon climate with moderate precipitation and high air humidity |
| 2400-2900 | Moderate temperate climate of the mountain | 8.0-12.0 | 2.0-5.0 | 14.0-17.0 | 2400-4200 | 1200-1300 | Humid monsoon climate, abundant rainfall, high air humidity, cool temperature, and humid climate |
| 2900-4000 | Cold temperate climate of the mountain | 2.5-8.0 | ＜-3.0-2.0 | ＜6.5-14.0 | ＜2400 | 1300-1500 | Humid monsoon climate, cold and humid climate with many clouds and fog |
| 4000-4344.1 | Sub-frigid climate of the mountain | 0-2.5 | -5.5--3.0 | 4-6.5 | N/A | 1500-1700 | Humid monsoon climate, very cold and humid climate with many clouds and fog |

**Table S2.** Vertical Soil taxonomy of Jiaozi Mountain National Natural Reserve (Based on Soil Classification Systems of China)^1^.

| **Altitude (m)** | **Soil Order** |  | **Soil Great Group** |
| --- | --- | --- | --- |
| 1100-1400 | Semi-alfisols |  | Torrid red soils |
| 1400-2300 | Ferrallisols |  | Red soils |
| 2300-2700 | Alfisols |  | Yellow soils |
| 2700-3300 | Alfisols |  | Brown soils |
| 3300-3700 | Alfisols |  | Dark brown soils |
| 3700-4000 | Alfisols |  | Brown coniferous forest soils |
| 4000-4344.1 | Alpine soils |  | Subalpine meadow soil |

**Table S3.** Vertical distribution of the main vegetation types on Jiaozi Mountain National Natural Reserve ^1^.

| **Altitude (m)** | **Vegetation Type** | **Main Vegetation** | **Area (hm^2^)** | **Proportion (%)** |
| --- | --- | --- | --- | --- |
| 1200-1500 | Hard-leaved evergreen oak forest in a dry and hot valley | *Quercus cocciferoides* | 263.29 | 1.60 |
| 2300-2600 | Semi-humid evergreen broad-leaved forest | *Schima argentea* | 57.82 | 0.35 |
|  |  | *Castanopsis orthacantha* | 234.10 | 1.42 |
| 2600-2900 | Wet evergreen broad-leaved forest in a medium height mountainous region | *Illicium simonsii* | 674.36 | 4.10 |
| 2600-3400 | Deciduous broad leaved forest | *Hydrangea macrophylla* | 199.33 | 1.21 |
|  |  | *Populus* spp.+*Betula* spp. | 67.96 | 0.41 |
| 2900-3500 | Mossy coppice at the top of the mountain | *Rhododendron* spp. | 1932.00 | 11.74 |
| 3000-3500 | Hard-leaved evergreen oak forest in cold temperate mountain area | *Quercus guyavifolia* | 1205.67 | 7.33 |
| 2700-3600 | Cool temperature coniferous forest | *Juniperus squamata* | 1050.50 | 6.38 |
|  |  | *Pinus densata* | 17.33 | 0.11 |
| 2800-3900 | Cold temperature coniferous forest | *Abies georgei var. smithii* | 1356.84 | 8.25 |
| 2600-4000 | Cold temperature shrub | *Rhododendron* spp. | 2357.01 | 14.32 |
|  |  | *Salix* spp. | 521.42 | 3.17 |
|  |  | *Juniperus* spp. | 839.38 | 5.10 |
|  |  | *Quercus* spp. | 1114.70 | 6.77 |
| 3100-3600 | Warm temperate bamboo forest | *Yushania violaxcens* | 227.20 | 1.38 |
| 2900—4200 | Cold-temperate meadow | Weed meadow | 3452.25 | 20.98 |
| 3920-4344.1 | Flowing rocky flat shrub | Mainly exposed flowing rocks |  |  |
| 2400-3000 | Artificial forest | *Pinus armandii* | 223.62 | 1.36 |
| Village, cultivated land, and wasteland | | | 661.50 | 4.02 |
| Summation | | | 16456.00 | 100.00 |

**Table S4.** Background information of the evaluators.

| **Information** | **Category** | **Number** | **Proportion (%)** |
| --- | --- | --- | --- |
| Gender | Men | 195 | 41.67 |
|  | Women | 273 | 58.33 |
| Age | Under 18 years old | 12 | 2.56 |
|  | 18-25 years old | 390 | 83.33 |
|  | 26-35 years old | 11 | 2.35 |
|  | 36-55 years old | 20 | 4.27 |
|  | 55 years old or older | 3 | 0.64 |
| Educational background | High school education and below | 12 | 2.56 |
|  | Vocational education | 26 | 5.56 |
|  | College | 306 | 65.38 |
|  | Postgraduate and above | 124 | 26.50 |
| Region origin | North China | 88 | 18.80 |
|  | Northeast China | 14 | 2.99 |
|  | Eastern China | 35 | 7.48 |
|  | Central China | 52 | 11.11 |
|  | South China | 17 | 3.63 |
|  | Northwest China | 35 | 7.48 |
|  | Southwest China | 227 | 48.50 |
| Professional background | landscape, forestry | 250 | 53.42 |
|  | Non-landscape, forestry | 238 | 50.85 |
| Summation | 468 | | |

**Table S5.** Autumnal variations in the color indices and scenic beauty estimation (SBE) value for the Jiaozi Mountain study site.

| **Index** | **Proportion of Five Color-changing Period (%)** | | | | | **F** | **P** |
| --- | --- | --- | --- | --- | --- | --- | --- |
|  | **DP1** | **DP2** | **DP3** | **DP4** | **DP5** |  |  |
| H1 | 0.177±0.0640 a | 0.2013±0.0680 a | 0.5106±0.1659 ab | 0.5629±0.1711 ab | 1.0504±0.3113 b | 3.274 | 0.014 |
| H2 | 0.328±0.1438 a | 0.4263±0.1139 a | 0.794±0.2256 ab | 1.0126±0.2664 ab | 1.8046±0.4018 b | 4.766 | 0.0014 |
| H3 | 2.4825±0.9912 a | 7.2072±1.6601 ab | 6.5544±1.1010 ab | 9.7572±1.8705 bc | 13.3154±2.4415 c | 5.201 | 0.0007 |
| H4 | 2.9514±0.9297 a | 10.441±2.4898 b | 7.9459±1.2102 b | 10.7702±1.8322 b | 8.8296±0.9054 b | 4.152 | 0.004 |
| H5 | 32.318±3.5331 a | 33.1531±3.3885 a | 32.2675±4.1174 a | 29.3607±3.0361 a | 25.8731±2.5235 a | 0.847 | 0.498 |
| H6 | 28.8111±2.6236 a | 12.4861±2.4118 b | 12.0658±1.9617 b | 10.9969±2.0687 b | 8.6357±1.3085 b | 15.015 | <0.001 |
| H7 | 9.4376±1.2913 a | 5.6113±1.6297 b | 3.6844±0.7873 bc | 3.192±0.7931 bc | 2.4321±0.4241 c | 8.173 | <0.001 |
| H8 | 4.2018±0.7493 a | 2.9719±0.9946 ab | 2.1216±0.5652 b | 1.6433±0.4199 b | 1.4285±0.2842 b | 3.665 | 0.008 |
| H9 | 3.3152±0.7656 a | 2.5847±0.8768 a | 2.207±0.6888 a | 1.986±0.4387 a | 2.3526±0.6154 a | 0.598 | 0.665 |
| H10 | 3.4198±1.1650 a | 2.7301±0.9910 a | 2.7982±0.9823 a | 5.2903±1.6808 a | 5.2542±1.2974 a | 0.956 | 0.435 |
| H11 | 0.4716±0.1321 a | 0.4294±0.1672 ab | 0.8486±0.2712 ab | 1.6211±0.6891 b | 1.6357±0.3160 b | 2.035 | 0.094 |
| H12 | 0.0379±0.0158 a | 0.0188±0.0077 ab | 0.0784±0.0239 ac | 0.0586±0.0170 abc | 0.0998±0.0196 c | 2.913 | 0.024 |
| H13 | 0.013±0.0054 a | 0.006±0.0031 ab | 0.0299±0.0096 ac | 0.0179±0.0050 abc | 0.0348±0.0097 c | 2.501 | 0.046 |
| H14 | 0.013±0.0052 a | 0.0059±0.0033 a | 0.027±0.0086 ab | 0.0158±0.0043 ab | 0.0376±0.0141 b | 2.047 | 0.092 |
| H15 | 0.0117±0.0043 a | 0.0072±0.0037 a | 0.0261±0.0071 ab | 0.0183±0.0051 a | 0.0468±0.0175 b | 2.494 | 0.047 |
| H16 | 0.0157±0.0051 a | 0.0125±0.0058 a | 0.0383±0.0097 ab | 0.0312±0.0088 a | 0.0782±0.0278 b | 2.917 | 0.024 |
| S1 | 43.4094±3.9419 a | 46.1123±3.7832 a | 44.9323±2.8937 a | 49.3788±3.7064 a | 52.7773±2.5200 a | 1.284 | 0.280 |
| S2 | 39.0091±3.3863 a | 26.8771±3.3349 b | 23.7293±3.8633 b | 22.8106±2.8505 b | 19.3382±3.2561 b | 5.151 | <0.001 |
| S3 | 4.9713±1.9045 a | 5.3035±2.0148 a | 3.3362±1.2608 a | 4.1456±1.9267 a | 0.7935±0.2572 a | 1.341 | 0.259 |
| White | 0.5627±0.1137 a | 1.2828±0.5750 a | 0.6068±0.1134 a | 0.8558±0.1613 a | 0.9191±0.2355 a | 1.076 | 0.372 |
| Gray | 6.0129±1.3667 a | 17.3288±4.3935 b | 24.113±3.9545 b | 17.8422±3.8403 b | 22.6908±3.2102 b | 4.046 | 0.004 |
| V1 | 40.7453±2.6219 a | 36.7995±2.9930 a | 39.8421±1.6046 a | 35.8919±2.3979 a | 39.4093±1.7419 a | 0.853 | 0.494 |
| V2 | 50.3204±2.8770 a | 55.987±3.2834 a | 54.4341±1.76446 a | 55.5539±2.7131 a | 54.651±2.2674 a | 0.718 | 0.581 |
| V3 | 2.8723±0.5956 a | 4.1178±1.0296 a | 2.4414±0.5389 a | 3.5871±0.4594 a | 2.4586±0.5722 a | 1.291 | 0.278 |
| Black | 6.062±1.1236 a | 3.0956±0.6331 a | 3.2824±0.7383 a | 4.9671±1.0614 a | 3.4812±0.7708 a | 1.891 | 0.117 |
| NC | 18±0.6887 a | 17.1053±0.9909 a | 17.3478±0.7240 a | 16.7857±0.8457 a | 17.2593±0.8210 a | 0.302 | 0.876 |
| MHI | 40.2811±2.5487 a | 35.0729±2.9323 a | 33.6485±3.9362 a | 33.6413±2.3618 a | 29.8917±2.3207 a | 1.799 | 0.134 |
| SBE value | 72.1335±4.72195 a | 81.1422±5.85113 a | 66.9146±4.64897 a | 48.1868±6.69268 b | 38.2317±5.54763 b | 9.529 | <0.001 |

Note: DP1: Pre-discoloration period, DP2: Early to middle discoloration period, DP3: Middle period of discoloration, DP4: Late period of discoloration, and DP5: End period of discoloration. Different letters indicate significant differences at the 0.05 level.

**Table S6.** Correlation matrix among SBE values and the 21 color indices significantly related to the SBE values.

|  | **SBE value** | **H1** | **H2** | **H3** | **H5** | **H6** | **H7** | **H8** | **H9** | **S1** | **S2** | **S3** | **Gray** | **V3** | **Black** | **NC** | **MHI** | **LPI** | **CON** | **DIV** | **SHDI** | **SIEI** |
| --- | --- | --- | --- | --- | --- | --- | --- | --- | --- | --- | --- | --- | --- | --- | --- | --- | --- | --- | --- | --- | --- | --- |
| SBE **value** | 1 |  |  |  |  |  |  |  |  |  |  |  |  |  |  |  |  |  |  |  |  |  |
| H1 | -0.462^***^ | 1 |  |  |  |  |  |  |  |  |  |  |  |  |  |  |  |  |  |  |  |  |
| H2 | -0.547^***^ | 0.93^***^ | 1 |  |  |  |  |  |  |  |  |  |  |  |  |  |  |  |  |  |  |  |
| H3 | -0.433 | 0.519^***^ | 0.744^***^ | 1 |  |  |  |  |  |  |  |  |  |  |  |  |  |  |  |  |  |  |
| H5 | 0.241^**^ | -0.334^***^ | -0.309^**^ | -0.118 | 1 |  |  |  |  |  |  |  |  |  |  |  |  |  |  |  |  |  |
| H6 | 0.414^***^ | -0.362^***^ | -0.447^***^ | -0.594 | 0.103 | 1 |  |  |  |  |  |  |  |  |  |  |  |  |  |  |  |  |
| H7 | 0.354^***^ | -0.295^**^ | -0.383^***^ | -0.53 | -0.227^*^ | 0.752^***^ | 1 |  |  |  |  |  |  |  |  |  |  |  |  |  |  |  |
| H8 | 0.312^**^ | -0.261^**^ | -0.346^***^ | -0.482 | -0.346^***^ | 0.517^***^ | 0.92^***^ | 1 |  |  |  |  |  |  |  |  |  |  |  |  |  |  |
| H9 | 0.237^**^ | -0.227^*^ | -0.311^**^ | -0.444 | -0.43^***^ | 0.262^**^ | 0.657^***^ | 0.863^***^ | 1 |  |  |  |  |  |  |  |  |  |  |  |  |  |
| S1 | -0.343^***^ | 0.082 | 0.112 | 0.069 | -0.197^*^ | -0.054 | 0.207^*^ | 0.304^**^ | 0.35^***^ | 1 |  |  |  |  |  |  |  |  |  |  |  |  |
| S2 | 0.495^***^ | -0.308^**^ | -0.278^**^ | -0.094 | 0.594^***^ | 0.48^***^ | 0.176 | -0.009 | -0.185^*^ | -0.56^***^ | 1 |  |  |  |  |  |  |  |  |  |  |  |
| S3 | 0.405^***^ | -0.169^*^ | -0.17 | -0.017 | 0.43^***^ | 0.026 | -0.126 | -0.178 | -0.212^*^ | -0.678^***^ | 0.505^***^ | 1 |  |  |  |  |  |  |  |  |  |  |
| Gray | -0.419^***^ | 0.324^***^ | 0.272^**^ | 0.077 | -0.612^***^ | -0.504^***^ | -0.344^***^ | -0.2^*^ | -0.028 | 0.023 | -0.784^***^ | -0.362^***^ | 1 |  |  |  |  |  |  |  |  |  |
| V3 | 0.27^**^ | -0.091 | -0.068 | 0.082 | 0.143 | -0.108 | -0.108 | -0.055 | 0.009 | -0.26^**^ | 0.212^*^ | 0.48^***^ | -0.219^*^ | 1 |  |  |  |  |  |  |  |  |
| Black | 0.339^***^ | -0.122 | -0.138 | -0.152 | 0.183^*^ | 0.327^***^ | 0.157 | 0.024 | -0.123 | -0.427^***^ | 0.495^***^ | 0.264^***^ | -0.45^***^ | 0.065 | 1 |  |  |  |  |  |  |  |
| NC | 0.442^***^ | -0.047 | -0.069 | -0.083 | 0.037 | 0.25^**^ | 0.365^***^ | 0.379^***^ | 0.342^***^ | -0.021 | 0.321^***^ | 0.181^*^ | -0.476^***^ | 0.286^**^ | 0.424^***^ | 1 |  |  |  |  |  |  |
| MHI | 0.214^*^ | -0.314^***^ | -0.262^**^ | -0.04 | 0.876^***^ | 0.212^**^ | -0.093 | -0.248^**^ | -0.378^***^ | -0.197^*^ | 0.695^***^ | 0.434^***^ | -0.699^***^ | 0.118 | 0.139 | -0.036 | 1 |  |  |  |  |  |
| LPI | -0.204^*^ | 0.097 | 0.118 | 0.164 | -0.23^*^ | -0.302^**^ | -0.281^**^ | -0.271^**^ | -0.217^**^ | -0.066 | -0.283^**^ | -0.068 | 0.421^***^ | 0.038 | -0.279^*^ | -0.387^***^ | -0.2^*^ | 1 |  |  |  |  |
| CON | -0.233^*^ | 0.101 | 0.106 | 0.148 | -0.263^**^ | -0.262^**^ | -0.185^*^ | -0.167 | -0.133 | 0.018 | -0.258^**^ | -0.118 | 0.363^***^ | -0.06 | -0.339^***^ | -0.399^***^ | -0.161 | 0.83^***^ | 1 |  |  |  |
| DIV | 0.22^*^ | -0.075 | -0.084 | -0.1 | 0.259^**^ | 0.265^**^ | 0.227^*^ | 0.212^*^ | 0.153 | 0.087 | 0.261^**^ | 0.062 | -0.409^***^ | -0.027 | 0.254^**^ | 0.372^***^ | 0.198^*^ | -0.962^***^ | -0.828^***^ | 1 |  |  |
| SHDI | 0.23^*^ | -0.226 | -0.261^**^ | -0.277 | 0.187^*^ | 0.399^***^ | 0.356^***^ | 0.309^**^ | 0.236^**^ | 0.123 | 0.242^**^ | -0.058 | -0.383^***^ | -0.099 | 0.294^**^ | 0.339^***^ | 0.154 | -0.84^***^ | -0.663^***^ | 0.844^***^ | 1 |  |
| SIEI | 0.23^*^ | -0.117 | -0.124 | -0.163 | 0.257^**^ | 0.28^**^ | 0.228^*^ | 0.226^*^ | 0.197^*^ | 0.029 | 0.26^**^ | 0.094 | -0.388^***^ | 0.023 | 0.312^**^ | 0.411^***^ | 0.165 | -0.92^***^ | -0.965^***^ | 0.923^***^ | 0.758^***^ | 1 |

**Table S7.** Differences in color indices of the five types of forest photos with superior visual aesthetic quality.

| **Index** | A | B | C | D | E | **F** | **P** |
| --- | --- | --- | --- | --- | --- | --- | --- |
| F1 | 0.71±0.14 a | 0.15±0.21 a | 0.36±0.26 a | -1.68 b | -0.13±0.22 a | 4.926 | 0.004 |
| F2 | 1.72±0.25 a | -0.48±0.12 b | 0.78±0.16 c | -1.06 b | -0.56±0.29 b | 27.7 | <0.001 |
| F3 | -0.03±0.15 a | 0.30±0.20 abe | 0.92±0.14 ce | -2.17 d | 0.95±0.09 bce | 18.411 | <0.001 |
| F4 | -0.79±0.04 a | 0.16±0.13 bc | -0.80±0.01 a | -0.09 c | 0.59±0.52 b | 24.783 | <0.001 |
| F5 | -0.46±0.15 a | 0.31±0.13 b | 1.61±0.09 c | 0.70 b | 1.90±0.06 c | 42.731 | <0.001 |
| F6 | -0.41±0.07 a | 0.23±0.22 a | 1.02±0.28 b | -0.49 a | 4.33±0.43 c | 19.203 | <0.001 |
| H1 | 0.03±0.01 a | 0.37±0.08 b | 0.02±0.01 a | 0.18 ab | 0.07 a | 8.201 | <0.001 |
| H2 | 0.06±0.03 a | 0.67±0.11 b | 0.04±0.01 a | 0.84 b | 0.34±0.07 a | 18.283 | <0.001 |
| H3 | 0.75±0.37 a | 10.45±1.36 b | 0.62±0.13 a | 7.66 b | 16.13±5.94 c | 23.473 | <0.001 |
| H5 | 19.58±2.10 a | 35.21±3.93 b | 39.97±3.53 b | 2.95 a | 39.77±7.62 b | 7.216 | <0.001 |
| H6 | 25.16±4.68 a | 7.46±1.72 b | 30.12±3.15 a | 1.42 b | 4.14±3.82 b | 14.374 | <0.001 |
| H7 | 14.72±2.48 a | 1.62±0.40 b | 6.49±0.98 c | 0.63 b | 0.85±0.84 b | 16.354 | <0.001 |
| H8 | 9.03±1.53 a | 0.87±0.19 b | 2.37±0.53 b | 0.91 b | 0.34±0.30 b | 18.081 | <0.001 |
| H9 | 8.72±1.52 a | 1.14±0.34 b | 1.65±0.44 b | 2.74 b | 0.38±0.31 b | 17.037 | <0.001 |
| S1 | 57.01±1.89 a | 43.17±1.33 b | 23.27±1.80 c | 30.59 c | 11.44±2.79 d | 63.872 | <0.001 |
| S2 | 29.60±2.77 a | 28.95±3.27 a | 48.42±3.28 b | 2.68 c | 46.65±6.97 b | 14.180 | <0.001 |
| S3 | 1.03±0.58 a | 3.70±1.11 a | 13.58±1.66 b | 0.00 a | 36.39±2.00 c | 36.973 | <0.001 |
| Gray | 6.76±1.60 a | 18.33±3.38 b | 1.95±0.33 a | 65.58 c | 1.25±0.31 a | 44.714 | <0.001 |
| V3 | 1.85±0.18 a | 3.70±1.05 a | 4.79±1.16 a | 1.90 a | 12.58±3.80 b | 4.364 | 0.008 |
| Black | 4.92±1.27 a | 5.27±1.70 a | 11.04±1.84 b | 0.00 a | 3.92±1.82 ab | 2.881 | 0.042 |
| NC | 20.33±1.12 a | 18.90±0.91 a | 18.30±1.02 a | 11.00 b | 22.50±1.50 a | 4.756 | 0.005 |
| MHI | 27.82±3.66 abd | 35.21±3.93 bc | 41.24±3.39 c | 10.92 d | 44.39±3.00 abc | 4.702 | 0.005 |
| LPI | 36.62±2.52 a | 51.75±4.36 b | 47.35±5.04 ab | 82.75 c | 55.29±7.47 abc | 4.634 | 0.006 |
| CON | 62.22±1.27 a | 64.68±2.02 a | 61.65±2.17 a | 78.32 b | 65.23±1.55 a | 3.395 | 0.023 |
| DIV | 0.77±0.03 a | 0.65±0.03 a | 0.68±0.05 a | 0.31 b | 0.65±0.08 a | 5.370 | 0.003 |
| SHDI | 1.67±0.14 a | 1.15±0.09 b | 1.33±0.20 ab | 0.59 bc | 1.02±0.03 abc | 2.692 | 0.053 |
| SIEI | 0.85±0.02 a | 0.74±0.04 a | 0.80±0.05 a | 0.39 b | 0.72 a | 5.501 | 0.002 |

Note: Different letters indicate significant differences at the 0.05 level.

**References**

1 Peng, H. & Liu, E. Yunnan Jiaozishan National Nature Reserve. 1-30，63-190，282-445 (China Forestry Publishing, 2015).
